# Kinetic resolution of racemic planar-chiral vinylcymantrenes by molybdenum-catalyzed asymmetric metathesis dimerization

**DOI:** 10.3762/bjoc.22.42

**Published:** 2026-03-31

**Authors:** Haruna Imazu, Hitoshi Izu, Yasuhiro Ohki, Masamichi Ogasawara

**Affiliations:** 1 Department of Natural Science, Graduate School of Science and Technology, Tokushima University, Minamijosanjima-cho, Tokushima 770-8506, Japan; 2 Institute for Chemical Research, Kyoto University, Uji 611-0011, Japan; 3 Tokushima International Science Institute, Tokushima University, Tokushima 770-8501, Japan

**Keywords:** cymantrene, kinetic resolution, molybdenum, olefin metathesis, planar chirality

## Abstract

Highly diastereo- and enantioselective kinetic resolution (KR) of a series of racemic planar-chiral 1-R-2-vinylcymantrenes (*rac*-**1**; R = Br, Me, I) was realized by an asymmetric metathesis dimerization (AMD) reaction catalyzed by a chiral molybdenum-alkylidene precatalyst. The Mo/(*R*)-**L1** precatalyst promoted the AMD/KR reaction of *rac*-**1a** (R = Br) to give (*E*)-(*R*,*R*)-**2a** of 99% ee together with unreacted recovered (*S*)-**1a** of 45% ee at 37% conversion. The diastereoselectivity of this reaction was excellent with *chiral*-**2a**/*meso*-**2a** = 96:4 molar ratio, and the selectivity factor (*k*_rel_) was calculated to be 754 based on a second-order equation. In all the three substrates examined, the dimerized products, *chiral*-**2**, were obtained in >98% ee thanks to the outstanding enantioselectivity.

## Introduction

The development of the well-defined molybdenum- [1–2] or ruthenium-alkylidene [3–5] catalysts has proven the olefin metathesis reaction to be a powerful tool in organic synthesis. Asymmetric extension is a recent trend in metathesis chemistry, and various chiral metal-alkylidene catalysts have been prepared over the past two decades [6–9].

Since 2002, our group and others have been interested in utilizing the olefin metathesis reaction for the modulation of various transition-metal complexes [10–13] thanks to the excellent tolerance of the Mo-/Ru-metathesis catalysts toward the organometallic substrates. The olefin metathesis protocols could be extended to the asymmetric synthesis of diverse planar-chiral transition metal complexes either by the kinetic resolution of the racemic substrates [14–16] or by the desymmetrization of the *C*_s_-symmetric substrates [17–19] by the use of an appropriate chiral catalyst (see the drawing in Table 1 for the structures of the representative chiral molybdenum precatalysts used in this study) [20–23]. Planar-chiral transition-metal complexes have been demonstrated to be excellent chiral scaffolds in asymmetric synthesis, however, enantioselective synthesis of such planar-chiral complexes is still relatively undeveloped and remains as a challenging problem in asymmetric synthesis [24–29].

**Scheme 1:**
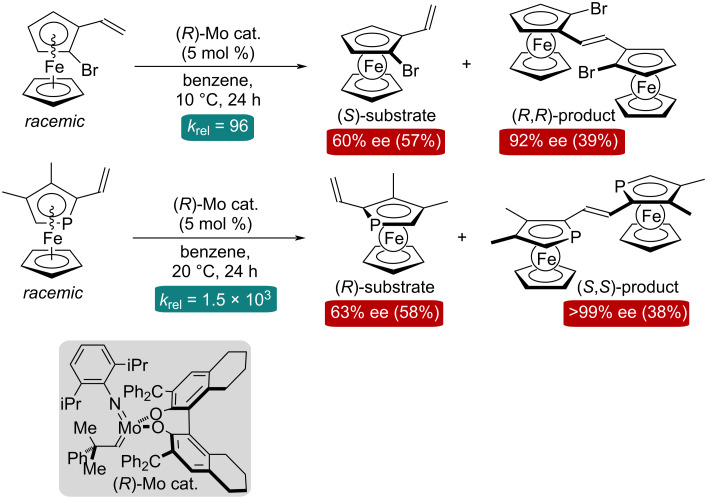
Asymmetric metathesis dimerization/kinetic resolution of racemic planar-chiral vinylferrocene/vinylphosphaferrocene.

A new type of asymmetric olefin metathesis reaction, that is the asymmetric metathesis dimerization (AMD; Scheme 1), was developed by our group during 2022–23 [30–31]. In the previous reports, racemic planar-chiral vinylferrocene [30] or vinylphosphaferrocene derivatives [31] were employed as substrates for the AMD process. In the presence of an appropriate chiral Mo-alkylidene precatalyst, the highly diastereo- and enantioselective kinetic resolution (KR) of the racemic substrates was attained with the *k*_rel_ values ([reaction rate of the fast-reacting enantiomer]/[reaction rate of the slow-reacting enantiomer]; selectivity factor) of up to 96 for the vinylferrocenes and of over 1000 for the vinylphosphaferrocenes.

In this article, we would like to report the analogous asymmetric metathesis dimerization/kinetic resolution of a series of racemic planar-chiral vinylcymantrenes (*rac*-**1a**–**c**). It was found that the chiral molybdenum-alkylidene precatalysts Mo/(*R*)-**L1** or Mo/(*R*)-**L3** discriminate the two enantiomers in *rac*-**1** efficiently to provide a mixture of the corresponding AMD product (*S*,*S*)-**2** and unreacted antipodal substrate (*R*)-**1** with the *k*_rel_ values of up to 1.5 × 10^3^. The AMD/KR reactions of *rac*-**1** were highly diastereoselective: under the optimized conditions, (*S*,*S*)-**2** was the sole AMD product and the formation of the respective mesomeric stereoisomer was negligible. It should be noted that this work is a rare example of catalytic asymmetric synthesis of planar-chiral CpMn(I) half-sandwich complexes [19,32].

## Results and Discussion

### Preparation of racemic planar-chiral 2-substituted vinylcymantrene substrates *rac*-**1a–c**

**Scheme 2:**
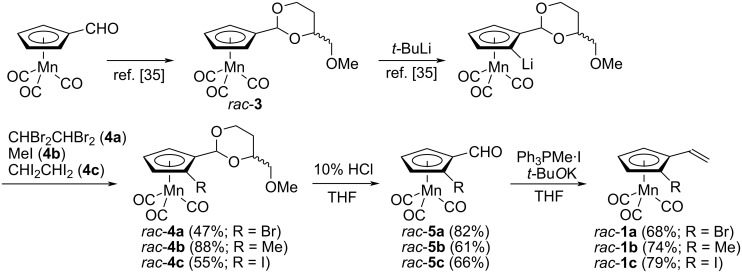
Preparation of racemic planar-chiral vinylcymantrene substrates *rac*-**1a**–**c**.

A series of racemic planar-chiral vinylcymantrene substrates *rac*-**1a**–**c** were prepared as outlined in Scheme 2. Whereas enantioselective synthesis of 2-substituted formylcymantrenes **5a**–**c**, precursors to **1a**–**c**, were reported [33–35], racemic **5a**–**c** were prepared in the same ways starting with *rac*-**3**. Vinylcymantrene substrates *rac*-**1a**–**c** were obtained in 68–79% yields by the Wittig methylenation of *rac*-**5a**–**c**. The moderate yields could be attributed to the volatility of *rac*-**1a**–**c**.

### Molybdenum-catalyzed asymmetric metathesis dimerization (AMD)/kinetic resolution (KR) of racemic planar-chiral vinylcymantrene derivatives *rac*-**1a–c**

The vinylcymantrene substrates, *rac*-**1**, prepared as above are planar-chiral due to the presence of an unsymmetrically substituted η^5^-(1-R-2-vinylcyclopentadienyl ligand (R = Br, Me, or I). The racemic substrates were examined in the AMD/KR studies, and the results are summarized in Table 1.

**Table 1:** Molybdenum-catalyzed asymmetric metathesis dimerization/kinetic resolution of racemic vinylcymantrenes **1a**–**c**.^a^

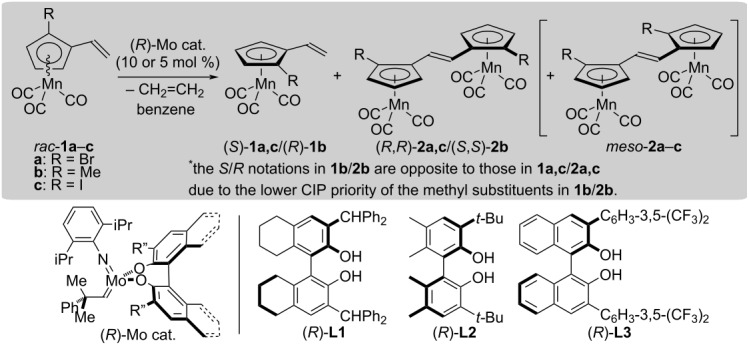									

Entry	Subst.	Chiral ligand	Conditions	Conversion		*chiral*-**2**/ *meso*-**2**^b^	% ee^d^		*k*_rel_^c^
	
				Exptl.^b^	Calcd.^c^		**1**	*chiral-* **2**	

1	*rac*-**1a**	(*R*)-**L1**	60 °C, 24 h	25%	26%	97:3	34	95	86

2	*rac*-**1a**	(*R*)-**L2**	60 °C, 24 h	3%	–^e^	–^e^	0.7	66	4.9

3	*rac*-**1a**	(*R*)-**L3**	60 °C, 24 h	51%	34%	85:15	48	95	107

4	*rac*-**1a**	(*R*)-**L1**	10 °C, 48 h	37%	31%	96:4	45	99	754

5	*rac*-**1a**	(*R*)-**L1**	10 °C, 72 h	18%	21%	98:2	26	97	120

6	*rac*-**1a**	(*R*)-**L3**	10 °C, 48 h	46%	42%	94:6	65	92	120

7	*rac*-**1a**	(*R*)-**L3**	10 °C, 72 h	23%	26%	96:4	33	92	47

8	*rac*-**1b**	(*R*)-**L1**	10 °C, 48 h	40%	32%	>99.5:<0.5	46	99	489

9	*rac*-**1b**	(*R*)-**L3**	10 °C, 48 h	58%	59%	73:27	74	88	21

10	*rac*-**1c**	(*R*)-**L1**	10 °C, 48 h	21%	29%	>99.5/<0.5	40	>99.5	1550

11	*rac*-**1c**	(*R*)-**L3**	10 °C, 48 h	29%	32%	>99.5:<0.5	47	98	286

12	*rac*-**1c**	(*R*)-**L3**	60 °C, 24 h	35%	29%	>99.5:<0.5	41	99	340

^a^The reaction was carried out with *rac*-**1** (0.20 mmol) in benzene (3.0 mL) for 24 h in the presence of a molybdenum catalyst generated in situ (10 mol %, except 5 mol % in entries 5 and 7); ^b^determined by the ^1^H NMR analysis of the crude reaction mixture; ^c^calculated from the % ee values of recovered **1** and *chiral*-**2** based on a second-order equation (refs. [37] and [38]; see Supporting Information File 1 for details); ^d^determined by chiral HPLC (see Supporting Information File 1 for details); ^e^not determined.

At the outset, the optimization of the reaction conditions, including a proper choice of chiral molybdenum-alkylidene precatalysts, was examined using *rac*-**1a** as a prototypical substrate. The AMD/KR reactions were conducted in benzene at the indicated temperature in the presence of an appropriate chiral Mo-precatalyst (10 mol %), which was generated in situ from the Mo-precursor, (pyrrolyl)_2_Mo(=CHCMe_2_Ph)(=N-C_6_H_3_-2,6-iPr_2_) [36], and an axially chiral biphenol ligand. The chiral Mo/(*R*)-**L1** precatalyst [21] facilitated the metathesis dimerization of (*R*)-**1a** preferentially, and the most of antipodal (*S*)-**1a** was recovered intact. In general, vinylcymantrenes are much less reactive than analogous vinylferrocenes in the molybdenum-catalyzed AMD [30], and the conversion tends to be lower. Only 25% conversion was attained in the reaction at 60 °C for 24 h. The crude reaction mixture was analyzed by ^1^H NMR measurement, which revealed the presence of the two diastereomers in the dimerized product with the molar ratio of *chiral*-**2a**/*meso*-**2a** = 97:3. The enantiomeric purity of recovered (*S*)-**1a** was determined to be 34% ee, while that of (*R*,*R*)-**2a** was found to be 95% ee (Table 1, entry 1). The *k*_rel_ value for the reaction was calculated to be 86 based on a second-order equation [30–31,37–38]. The Mo/(*R*)-**L2** precatalyst [22] was much less active than Mo/(*R*)-**L1** and less than 3% conversion was attained under the otherwise identical conditions (Table 1, entry 2). The Mo/(*R*)-**L3** precatalyst [23] showed the highest catalytic activity among examined. Although the enantioselectivity was excellent (*k*_rel_ = 107), the diastereoselectivity was poor (Table 1, entry 3). At 10 °C using Mo/(*R*)-**L1**, enantioselectivity was greatly improved in the AMD/KR of *rac*-**1a** giving (*R*,*R*)-**2a** of 99% ee and (*S*)-**1a** of 45% ee with 37% conversion (*k*_rel_ = 754; Table 1, entry 4). The lower catalyst loading (5 mol %) led to an unsatisfactory conversion (18%) probably due to the decomposition of the molybdenum catalyst prior to the completion of the reaction (Table 1, entry 5). On the other hand, the effects of lowering the temperature were minimal in the reactions using Mo/(*R*)-**L3** (Table 1, entries 6 and 7).

The optimized conditions as in entries 4 and 6 were applied to the AMD/KR reactions of *rac*-**1b** and **1c** as well. The AMD/KR of *rac*-**1b** using Mo/(*R*)-**L1** proceeded with excellent diastereo-/enantioselectivities (*chiral*-**2b**/*meso*-**2b** = >99.5:<0.5; *k*_rel_ = 489), and (*R*)-**1b** and (*S*,*S*)-**2b** were obtained in 46% ee and 99% ee, respectively (Table 1, entry 8). On the other hand, the results using Mo/(*R*)-**L3** were poor with *chiral*-**2b**/*meso*-**2b** = 73:27 and *k*_rel_ = 21 (Table 1, entry 9). The AMD reaction of *rac*-**1c** was sluggish. The conversion at 10 °C in 48 h was only 21% using Mo/(*R*)-**L1** (*k*_rel_ = 1.5 × 10^3^; *chiral*-**2c**/*meso*-**2c** = >99.5:<0.5; Table 1, entry 10) and 29% using Mo/(*R*)-**L3** (*k*_rel_ = 286; *chiral*-**2c**/*meso*-**2c** = >99.5:<0.5; Table 1, entry 11) despite the excellent enantio- and diastereoselectivities. The conversion was improved to 35% at 60 °C using Mo/(*R*)-**L3**, and the excellent enantio- and diastereoselectivities were retained (*k*_rel_ = 340; *chiral*-**2c**/*meso*-**2c** = >99.5:<0.5; Table 1, entry 12). All the AMD products were obtained in *E*-configurations exclusively.

### Determination of the absolute configuration of (–)-**2b**

**Figure 1:**
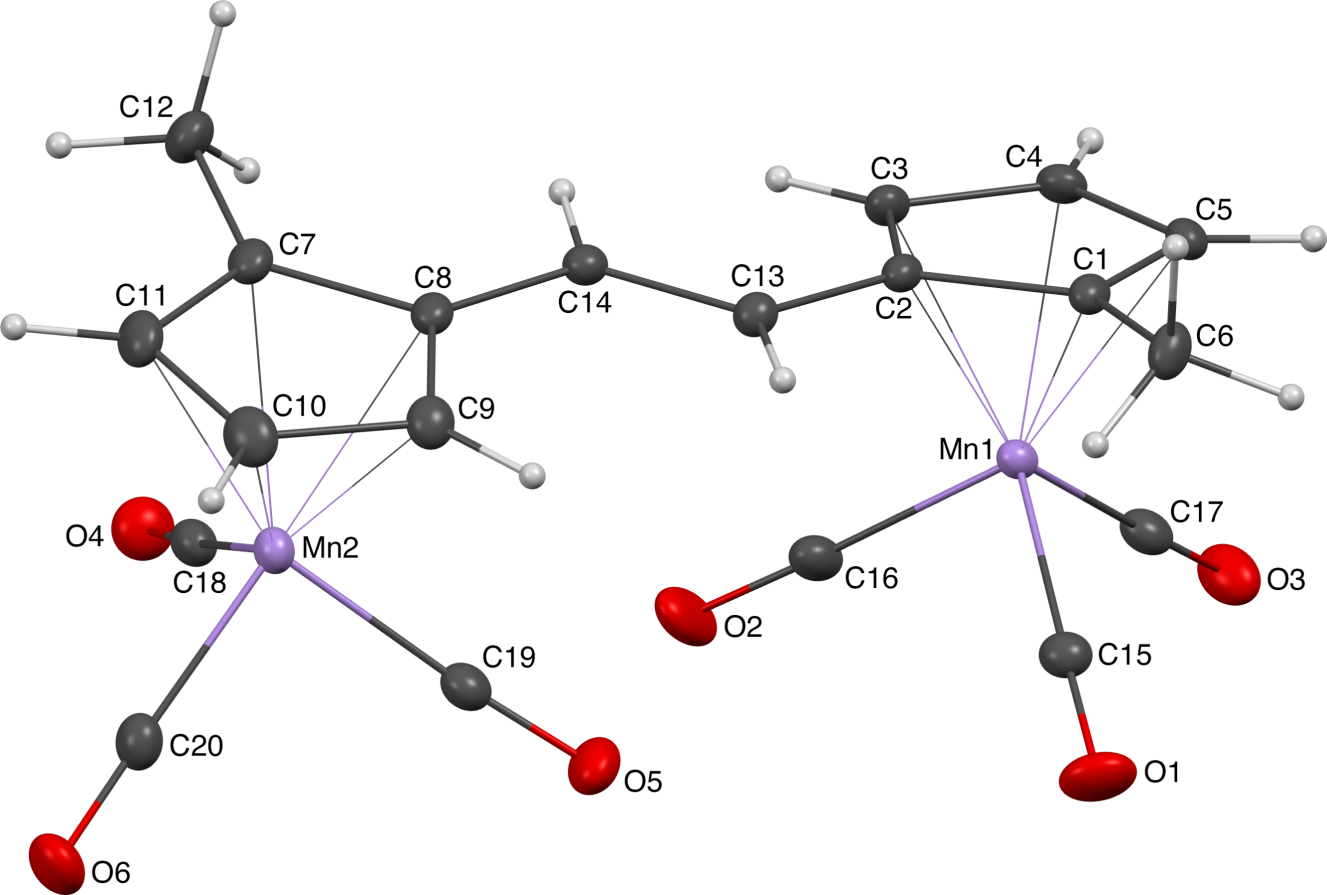
ORTEP drawing of the X-ray structure of (*S*,*S*)-(–)-**2b** with atom numbering (thermal ellipsoids set at the 30% probability level). Selected bond lengths (Å) and angles (deg): C2–C13 = 1.460(3), C8–C14 = 1.461(3), C13–C140 = 1.340(3), Mn1-least-squares plane_C1–C5_ = 1.771 (7), Mn2-least-squares plane_C7–C11_ = 1.770(7); C2–C13–C14 = 123.6(2), C8–C14–C13 = 125.7(2), dihedral angle between least-squares plane_C1–C5_ and least-squares plane_C7–C11_ = 17.40(8).

Levorotatory AMD/KR product (–)-**2b**, which was obtained as in entry 8, Table 1 using Mo/(*R*)-**L1** precatalyst, was recrystallized by slow diffusion of pentane into the concentrated diethyl ether solution. Crystals of (–)-**2b** were grown as light-yellow blocks. The X-ray crystallography revealed that the unit cell contains two independent molecules, having slightly different conformations, and the structure of one of the two crystallographically independent molecules is shown in Figure 1 with the selected bond lengths and angles (see Supporting Information File 1 and Supporting Information File 2 for details).

The two cyclopentadienides and the olefinic moiety are nearly coplanar with a C2–C13–C14–C8 torsion angle of 175.4(2)°. The Flack parameter for this structure was determined to be –0.010(4), and the absolute configuration of (–)-**2b** was unambiguously determined to be (*S*,*S*) (see Supporting Information File 1 and Supporting Information File 2 for details).

### Comparison of vinylcymantrenes, vinylferrocenes, and vinylphosphaferrocenes in molybdenum-catalyzed AMD/KR

**Figure 2:**
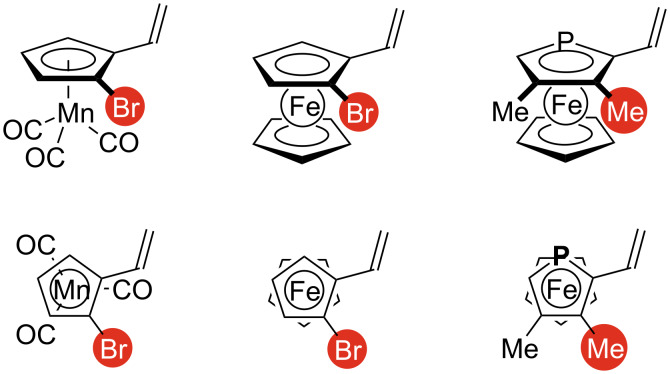
Structures of less-reactive enantiomers in three representative planar-chiral vinylmetallocene substrates in Mo/(*R*)-**L1**-catalyzed AMD/KR.

Figure 2 shows the structures of the less-reactive enantiomers in the three representative substrates (vinylcymantrene, vinylferrocene [30], and vinylphosphaferrocene [31]) in the AMD/KR reactions using Mo/(*R*)-**L1**. The views of the molecules from the view point opposite to the central metal cations (Mn(I) or Fe(II)) are illustrated at the bottom of Figure 2. All the three compounds possess the similar structural features: (1) presence of a substituent (Br or Me) at the position adjacent to the vinyl group in the clockwise direction, (2) absence of a substituent other than hydrogen at the position adjacent to the vinyl group in the counterclockwise direction (CH or P). The substituent adjacent to the vinyl group (marked in red in Figure 2) likely inhibits the effective interaction of the substrate with the chiral catalyst, resulting in highly enantioselective kinetic resolution.

Cymantrene is far less electron-poor than ferrocene due to the presence of the three carbonyl ligands, which are strong π-acids, on the manganese(I). Indeed, the Friedel–Crafts acetylation of cymantrene, a typical electrophilic aromatic substitution reaction, is much slower than that of ferrocene. Consequently, vinylcymantrenes are electron-deficient olefins and less reactive in olefin metathesis. For this reason, the present AMD/KR reactions of *rac*-**1a**–**c** require a relatively high catalyst loading, resulting in lower conversions.

## Conclusion

In summary, we have developed a protocol for the kinetic resolution (KR) of racemic planar-chiral 1-R-2-vinylcymantrenes (*rac*-**1**; R = Br, Me, I) by the molybdenum-catalyzed asymmetric metathesis dimerization (AMD). The AMD/KR reactions of *rac*-**1** proceeded with near-perfect diastereoselectivity, of which enantioselectivity was also excellent with the *k*_rel_ values of up to 1.5 × 10^3^. Because of the outstanding enantioselectivity, dimerized products *chiral*-**2** were obtained in very high enantiomeric purity of up to >99% ee. Vinylcymantrenes are electron-deficient olefins, and they are poorer substrates in olefin metathesis. For this reason, the AMD/KR reactions of *rac*-**1a**–**c** require a relatively high catalyst loading and the conversions tend to be lower.

This study, together with our previous reports [29], reveals that the molybdenum-catalyzed asymmetric metathesis reactions are powerful tools to control planar chirality in various transition-metal complexes.

## Supporting Information

File 1 Experimental procedures, NMR spectra (^1^H and ^13^C) for all the new compounds, and chiral HPLC chromatograms.

File 2 Crystallographic information file for compound **2b**.

## Data Availability

All data that supports the findings of this study is available in the published article and/or the supporting information of this article.
