# Supplementary material for: Kinetic resolution of racemic planar-chiral vinylcymantrenes by molybdenum-catalyzed asymmetric metathesis dimerization
Source: Beilstein J Org Chem. 2026 Mar 31;22:568–74. doi: 10.3762/bjoc.22.42 (PMC13058277; doi:10.3762/bjoc.22.42)
Supplement: File 1 — Experimental procedures, NMR spectra (1H and 13C) for all the new compounds, and chiral HPLC chromatograms. [file Beilstein_J_Org_Chem-22-568-s001.pdf]

BEILSTEIN JOURNAL OF ORGANIC CHEMISTRY

Supporting Information

for

Kinetic resolution of racemic planar-chiral vinylcymantrenes by molybdenum-catalyzed asymmetric metathesis dimerization

Haruna Imazu, Hitoshi Izu, Yasuhiro Ohki and Masamichi Ogasawara

Beilstein J. Org. Chem. **2026,** *22,* 568–574. doi:10.3762/bjoc.22.42

Experimental procedures, NMR spectra (^{1}H and ^{13}C) for all the new compounds, and chiral HPLC chromatograms

License and Terms: This is a supporting information file under the terms of the Creative Commons Attribution License (https://creativecommons.org/licenses/by/4.0). Please note that the reuse, redistribution and reproduction in particular requires that the author(s) and source are credited and that individual graphics may be subject to special legal provisions.
The license is subject to the *Beilstein Journal of Organic Chemistry* terms and conditions: (https://www.beilstein-journals.org/bjoc/terms)

Table of contents

Experimental section. S2–S3
References. S4
^{1}H and ^{13}C NMR spectra of vinylcymantrenes **1a–c**. S5–S10
^{1}H and ^{13}C NMR spectra of 1,2-dicymantrenylethylenes **2a**–**c** S11–S16
Chiral HPLC chromatograms of **1a–c** and **2a–c** (Table 1; entries 4, 8, and 10). S17–S22

Experimental section.

General information. All anaerobic and/or moisture sensitive manipulations were carried out with standard Schlenk techniques under predried nitrogen or with glovebox techniques under prepurified argon. ^{1}H NMR (at 400 MHz) and ^{13}C NMR (at 101 MHz) chemical shifts are reported in ppm downfield of internal tetramethylsilane. Tetrahydrofuran and benzene were distilled from benzophenone-ketyl under nitrogen prior to use. Dichloromethane was distilled from CaH_2 under nitrogen prior to use. C_6D_6 was distilled from Na/K under vacuum and stored in a glovebox. Following compounds were prepared as reported: *rac*-1-bromo-2-formylcymantrene (*rac*-**5a**) [S1], *rac*-2-methyl-1-formylcymantrene (*rac*-**5b**) [S2], *rac*-2-iodo-1-formylcymantrene (*rac*-**5c**) [S3], (pyrrolyl)$_2$Mo(=CHCMe$_2$Ph)(=N-C$_6$H$_3$-2,6-^{i}Pr$_2$) [S4], (*R*)-3,3'-(Ph$_2$CH)$_2$-H$_8$-binaphthol (**L1**) [S5], (*R*)-3,3'-[3,5-(CF$_3$)$_2$C$_6$H$_3$]$_2$-2,2'-binaphthol (**L3**) [S6]. All the other chemicals were obtained from commercial sources and used as received unless otherwise noted.

***rac*-1-Bromo-2-vinylcymantrene (*rac*-1a).** Methyltriphenylphosphonium iodide (1.08 g, 2.67 mmol) and ^{t}BuOK (299 mg, 2.66 mmol) were suspended in dry THF (20 mL) and the suspension was stirred for 15 min at room temperature. To this was added a THF (3 mL) solution of *rac*-1-bromo-2-formylcymantrene (*rac*-**5a**, 921 mg, 2.96 mmol) dropwise at room temperature. After stirring the mixture for 4 h, the reaction mixture was extracted with hexane. The organic extracts were combined and filtered through a pad of silica gel. The filtrate was evaporated to dryness. The residue was chromatographed on silica gel (hexane/benzene = 4/1) to give *rac*-**1a** as a yellow oil. Yield: 620 mg, (68%). ^{1}H NMR (400 MHz, C_6D_6): δ 6.14 (dd, *J* = 17.5 and 11.0 Hz, 1H), 5.15 (d, *J* = 17.5 Hz, 1H), 4.89 (d, *J* = 11.0 Hz, 1H), 4.09−4.08 (m, 1H), 4.04−4.03 (m, 1H), 3.77−3.75 (m, 1H). ^{13}C{^{1}H} NMR (101 MHz, C_6D_6): δ 224.4, 127.2, 117.1, 99.3, 86.8, 82.5, 82.1, 76.5. HRMS (ESI) m/z: [M + H]$^+$ Calcd for $C_{10}H_7MnBrO_3^+$: 308.8954. Found: 308.8959.

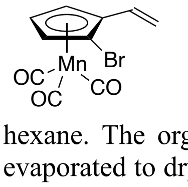

***rac*-1-Methyl-2-vinylcymantrene (*rac*-1b).** This compound was prepared in the same way with *rac*-**1a** starting with methyltriphenylphosphonium iodide (4.17 g, 10.3 mmol), ^{t}BuOK (1.11 g, 9.89 mmol), and *rac*-2-methyl-1-formylcymantrene (*rac*-**5b**, 1.94 g, 7.88 mmol). The crude compound was chromatographed on silica gel (hexane/dichloromethane = 3/1) to give *rac*-**1b** as a yellow oil. Yield:1.43 g, (74%). ^{1}H NMR (400 MHz, C_6D_6): δ 5.90 (dd, *J* = 17.5 and 11.0 Hz, 1H), 5.12 (d, *J* = 17.5 Hz, 1H), 4.87 (d, *J* = 11.0 Hz, 1H), 4.27 (m, 1H), 3.96−3.95 (m, 1H), 3.78 (s, 1H), 1.48 (s, 1H). ^{13}C{^{1}H} NMR (101 MHz, C_6D_6): δ 223.7, 126.0, 113.3, 99.4, 96.0, 79.5, 79.2, 76.7, 9.9. HRMS (ESI) m/z: [M + H]$^+$ Calcd for $C_{11}H_{10}MnO_3^+$: 245.0005 Found: 245.0018.

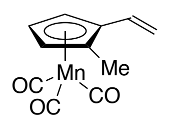

***rac*-1-Iodo-2-vinylcymantrene (*rac*-1c).** This compound was prepared in the same way with *rac*-**1a** starting with methyltriphenylphosphonium iodide (820 mg, 2.03 mmol), ^{t}BuOK (228 mg, 2.03 mmol), and *rac*-2-iodo-1-formylcymantrene (*rac*-**5c**, 760 mg, 2.13 mmol). The crude compound was chromatographed on silica gel (hexane/diethyl ether = 9/1) to give *rac*-**1b** as a yellow solid. Yield: 598 mg, (79%). ^{1}H NMR (400 MHz, C_6D_6): δ 6.08 (dd, *J* = 17.5 and 10.9 Hz, 1H), 5.11 (dd, *J* = 17.5 and 0.7 Hz, 1H), 4.87 (dd, *J* = 10.9 and 0.9 Hz, 1H), 4.17−4.15 (m, 1H), 4.07−4.06 (m, 1H), 3.83−3.82 (m, 1H). ^{13}C{^{1}H} NMR (101 MHz, C_6D_6): δ 224.7, 129.4, 117.3, 103.3, 88.2, 84.4, 77.2, 49.6. HRMS (ESI) m/z: [M] Calcd for $C_{10}H_6MnIO_3$: 355.8742. Found: 355.8754.

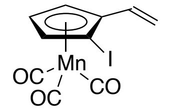

General procedure for the molybdenum-catalyzed asymmetric metathesis dimerization/kinetic resolution of 1a–c. The reaction conditions and the results are summarized in Table 1. The reactions were conducted in a manner similar to ref.-S7. In a glovebox under purified argon, Mo(=CHCMe$_2$Ph)(=NC$_6$H$_3$-2,6-^{i}Pr$_2$)(NC$_4$H$_4$)$_2$, (12.2 mg, 22.8 μmol) and (*R*)-3,3'-(Ph$_2$CH)$_2$-H$_8$-2,2'-binaphthol (**L1**; 14.3 mg, 22.8 μmol) were dissolved in dry benzene (1.0 mL) in a 20 mL Schlenk flask. After stirring the solution for 15 min at room temperature, to this was added a solution of *rac*-**1a** (70.0 mg, 227 μmol) in benzene (2.5 mL). The Schlenk flask was sealed tightly and taken out of the glovebox. The mixture was stirred for 48 h at the given temperature. After quenching the reaction by the addition of acetone (ca. 100 μL), the reaction mixture was evaporated to dryness under reduced pressure. The conversion of the AMD/kinetic resolution reaction was determined by the ^{1}HNMR analysis of the crude product. The crude product was chromatographed on silica gel (hexane/dichloromethane = 3/1) under nitrogen to give remaining **1a** (23.6 mg, 34%) and AMD product **2a** (26.6 mg, 38%). The characterization data of **2a–c** and the conditions for chiral HPLC analysis of **1a–c** and **2a–c** are listed below.

(*R*,*R*)-(*E*)-1,2-Di(2-bromocymantrenyl)ethylene (*chiral*-2a). ^{1}H NMR (400 MHz, C_6D_6): δ 6.32 (s, 2H), 4.09−4.07 (m, 2H), 3.95−3.94 (m, 2H), 3.74−3.73 (m, 2H). ^{13}C{^{1}H} NMR (101 MHz, C_6D_6): δ 224.0, 121.8, 97.4, 87.5, 82.6, 82.3, 77.2. HRMS (ESI) m/z: $[M + H]^+$ Calcd for $C_{18}H_9Mn_2Br_2O_6^+$: 590.7501. Found: 590.7496. $[\alpha]^{28}_D$ = −371 (*c* 2.41, CH_2Cl_2, for (*R*,*R*)-isomer of >99% ee). Chiral HPLC analysis conditions: Chiralpak IE; eluent: hexane/ethyl acetate/^{i}PrOH = 90/5/1; flow rate: 0.5 mL/min; t_1 = 11.6 min ((*S*,*S*)-isomer), t_2 = 12.1 min ((*R*,*R*)-isomer).

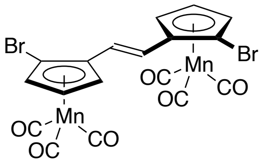

(*S*)-1-Bromo-2-vinylcymantrene ((*S*)-1a). $[\alpha]^{27}_D$ = +85 (*c* 1.34, CH_2Cl_2, 63% ee). Chiral HPLC analysis conditions: Chiralcel OD-H; eluent: hexane/^{i}PrOH = 50/1; flow rate: 1.0 mL/min; t_1 = 35.4 min ((*S*)-isomer), t_2 = 43.0 min ((*R*)-isomer).

(*S*,*S*)-(*E*)-1,2-Di(2-methylcymantrenyl)ethylene (*chiral*-2b). ^{1}H NMR (400 MHz, C_6D_6): δ 6.07 (s, 2H), 4.24−4.23 (m, 2H), 3.97−3.95 (m, 2H), 3.80−3.79 (m, 2H), 1.56 (s, 6H). ^{13}C{^{1}H} NMR (101 MHz, C_6D_6): δ 225.4, 121.4, 101.4, 97.1, 81.6, 81.5, 79.3, 12.1. HRMS (ESI) m/z: $[M]^+$ Calcd for $C_{20}H_{14}Mn_2O_6^+$: 459.9546. Found: 459.9543. $[\alpha]^{28}_D$ = −391 (*c* 2.32, CH_2Cl_2, for (*S,S*)-isomer of 93% ee). Chiral HPLC analysis conditions: Chiralpak IC; eluent: hexane/^{i}PrOH = 100/1; flow rate: 0.5 mL/min; t_1 = 13.8 min ((*R*,*R*)-isomer), t_2 = 14.9 min ((*S*,*S*)-isomer).

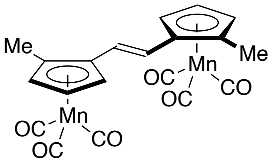

(*R*)-1-Methyl-2-vinylcymantrene ((*R*)-1b). $[\alpha]^{23}_D$ = +113 (*c* 4.89, CH_2Cl_2, 62% ee). Chiral HPLC Analysis Conditions: Chiralcel OD-H; eluent: hexane/^{i}PrOH = 10/1; flow rate: 0.5 mL/min; t_1 = 45.9 min ((*R*)-isomer), t_2 = 49.9 min ((*S*)-isomer).

(*R*,*R*)-(*E*)-1,2-Di(2-iodocymantrenyl)ethylene (*chiral*-2c). ^{1}H NMR (400 MHz, C_6D_6): δ 6.24 (s, 2H), 4.15 (m, 2H), 4.00 (m, 2H), 3.80−3.79 (m, 2H). ^{13}C{^{1}H} NMR (101 MHz, C_6D_6): δ 224.4, 124.1, 101.3, 88.3, 84.8, 78.0, 50.6. HRMS (ESI) m/z: $[M + H]^+$ Calcd for $C_{18}H_9Mn_2I_2O_6^+$: 684.7244. Found: 684.7238. $[\alpha]^{29}_D$ = −172 (*c* 2.17, CH_2Cl_2, for (*R*,*R*)-isomer of 99% ee). Chiral HPLC analysis conditions: Chiralpak IB; eluent: hexane/ethyl acetate/^{i}PrOH = 90/5/1; flow rate: 0.5 mL/min; t_1 = 21.2 min ((*S*,*S*)-isomer), t_2 = 22.2 min ((*R*,*R*)-isomer).

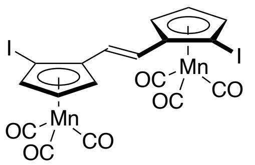

(*S*)-1-Iodo-2-vinylcymantrene ((*S*)-1c). $[\alpha]^{28}_D$ = +124 (*c* 3.91, CH_2Cl_2, 40% ee). Chiral HPLC Analysis Conditions: Chiralpak IB; eluent: hexane/ethyl acetate/^{i}PrOH = 900/60/1; flow rate: 0.5 mL/min; t_1 = 12.1 min ((*S*)-isomer), t_2 = 12.6 min ((*R*)-isomer).

X-ray crystallographic structure determinations. Single crystal X-ray diffraction data of (*S*,*S*)-**2b** was collected on a Rigaku RA-Micro 7 equipped with a Rigaku HyPix-6000HE detector that uses mirror-monochromated MoKα radiation (λ = 0.71073 Å) at –150 °C under a cold N_2 stream. Single crystals of the compound were coated with oil (Immersion Oil, Type B: code 1248, Cargille Laboratories, Inc.) and placed on Polyimide crystal mounts. The crystal quality and preliminary cell parameters were determined by eighteen data frames measured at 0.5° increments of ω. Afterward, the full data sets were also measured at 0.5° intervals of ω. Each frame of data was integrated using the CrysAlisPro program package [S8], followed by the data correction for absorption using an REQAB program. The structure was solved with Intrinsic Phasing using SHELXT [S9] and refined by full-matrix least-square on F^2 using SHELXL [S10] in Olex2 [S11]. Anisotropic refinement was applied to all non-hydrogen. Hydrogen atoms were placed using a riding model. The absolute structure was determined by refinement of the Flack parameter using the method of Parsons, Flack, and Wagner based on quotients of Friedel pairs [S12].

Crystallographic data have been deposited with Cambridge Crystallographic Data Centre. Deposition numbers CCDC 2525244 (for (*S*,*S*)-**2b**) contain the supplementary crystallographic data for this paper. This data is provided free of charge by the joint Cambridge Crystallographic Data Centre and Fachinformationszentrum Karlsruhe Access Structures service.

References.

[S1] Kamikawa, K.; Tseng, Y.-Y.; Jian, J.-H.; Takahashi, T.; Ogasawara, M. *J. Am. Chem. Soc.* **2017**, *139*, 1545−1553.

[S2] Loim, N. M.; Kondratenko, M. A.; Sokolov, V. I. *J. Org. Chem.* **1994**, *59*, 7485–7487.

[S3] Ferber, B.; Top, S.; Jaouen, G. *J. Organomet. Chem.* **2004**, *689*, 4872−4876.

[S4] Hock, A. S.; Schrock, R. R.; Hoveyda, A. H. *J. Am. Chem. Soc.* **2006**, *128*, 16373−16375.

[S5] Schrock, R. R.; Jamieson, J. Y.; Dolman, S. J.; Miller, S. A.; Bonitatebus, Jr., P. J.; Hoveyda, A. H. *Organometallics* **2002**, *21*, 409−417.

[S6] Singh, R.; Czekelius, C.; Schrock, R. R.; Müller, P.; Hoveyda, A. H. *Organometallics* **2007**, *26*, 2528−2539.

[S7] Masaoka, K.; Taue, H.; Wakioka, M.; Ohki, Y.; Ogasawara, M. *Organometallics* **2023**, *42*, 1629−1638.

[S8] Rigaku Oxford Diffraction, CrysAlisPrO, version 1.171.43.130a, Rigaku Corporation, Oxford, UK, **2024**)

[S9] Sheldrick, G. M. *Acta Cryst.* **2015**, *A71*, 3−8.

[S10] Sheldrick, G. M. *Acta Cryst.* **2015**, *C71*, 3−8.

[S11] Dolomanov, O. V.; Bourhis, L. J.; Howard, J. A. K.; Puschmann, H. *Acta Cryst.* **2009**, *42*, 339−341.

[S12] Parsons, S.; Flack, H. D.; Wagner, T. *Acta Cryst.* **2013**, *B69*, 249−259.

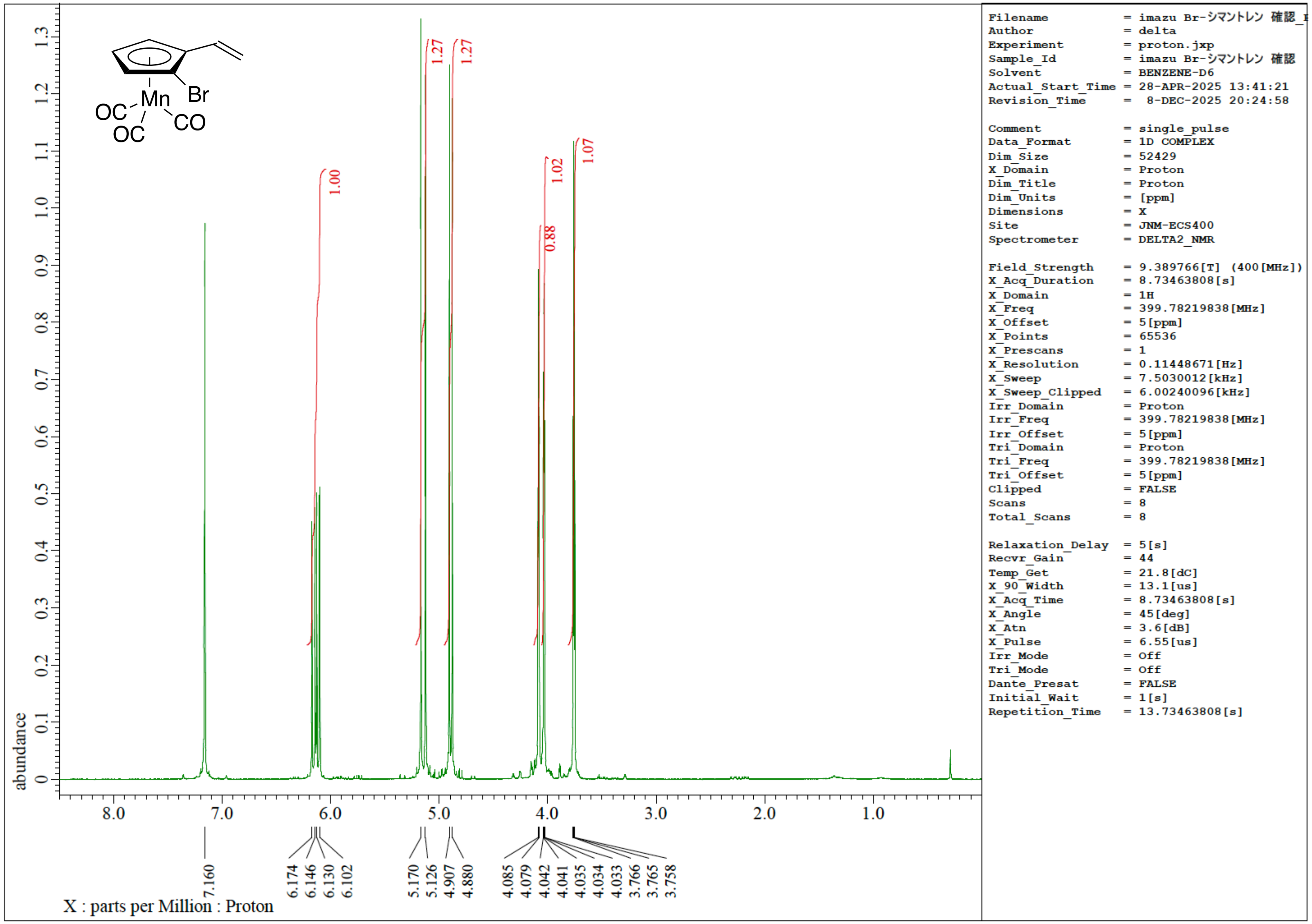

Figure S1. ^{1}H NMR Spectrum of **1a** in C_6D_6 at 400 MHz.

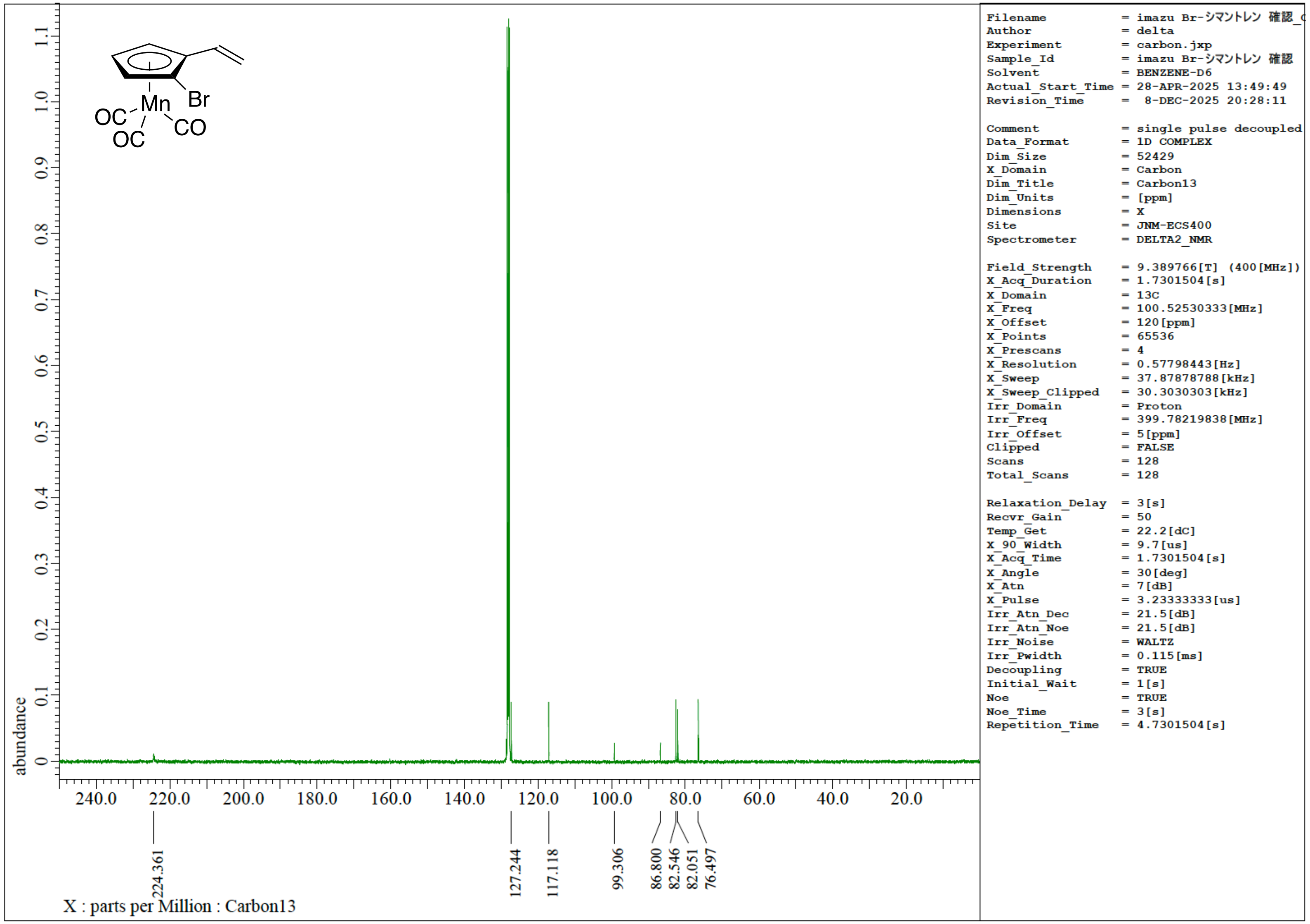

Figure S2. ^{13}C NMR Spectrum of **1a** in C_6D_6 at 101 MHz.

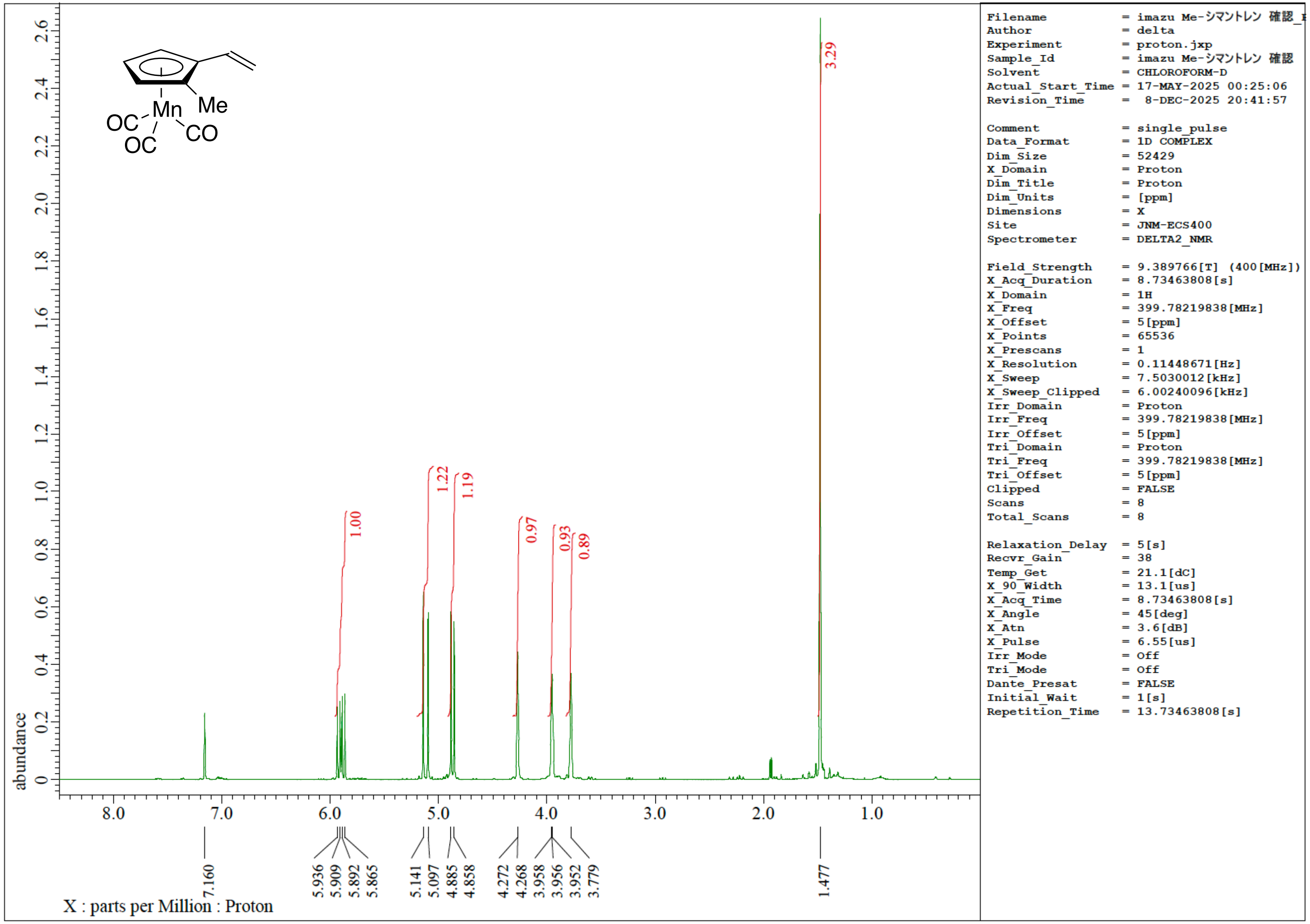

Figure S3. ^{1}H NMR Spectrum of **1b** in C_6D_6 at 400 MHz.

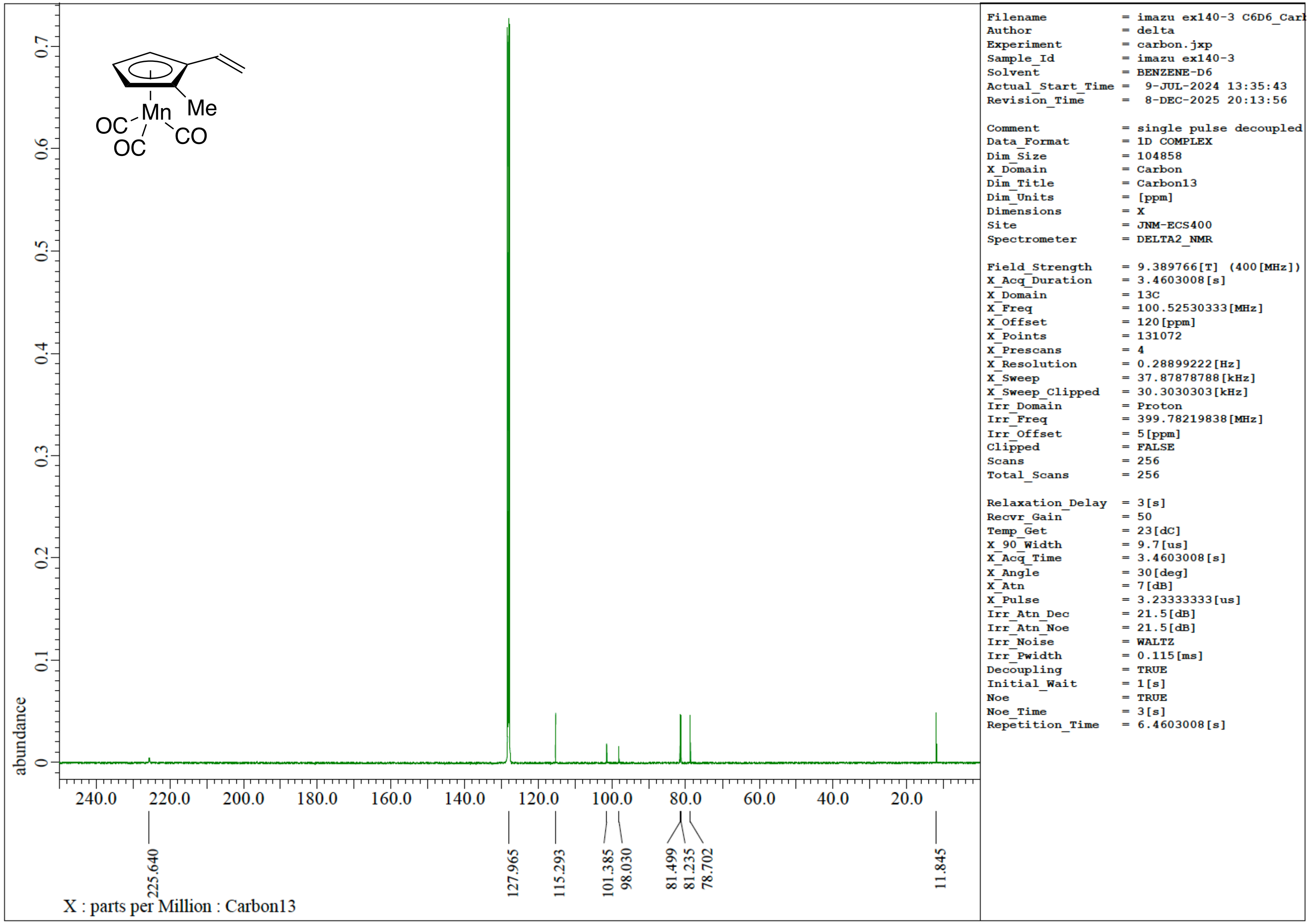

Figure S4. ^{13}C NMR Spectrum of **1b** in C_6D_6 at 101 MHz.

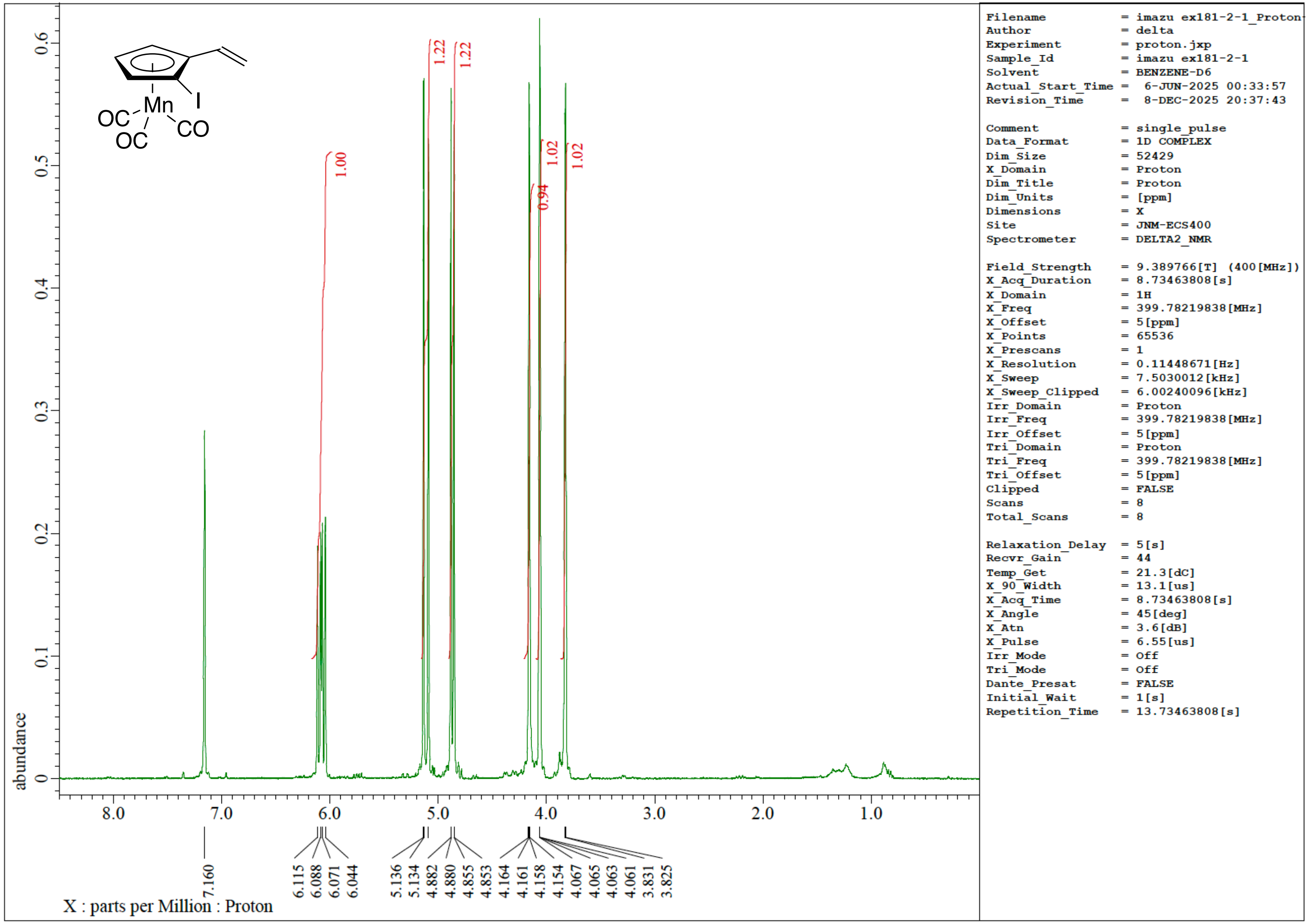

Figure S5. ^{1}H NMR Spectrum of **1c** in C_6D_6 at 400 MHz.

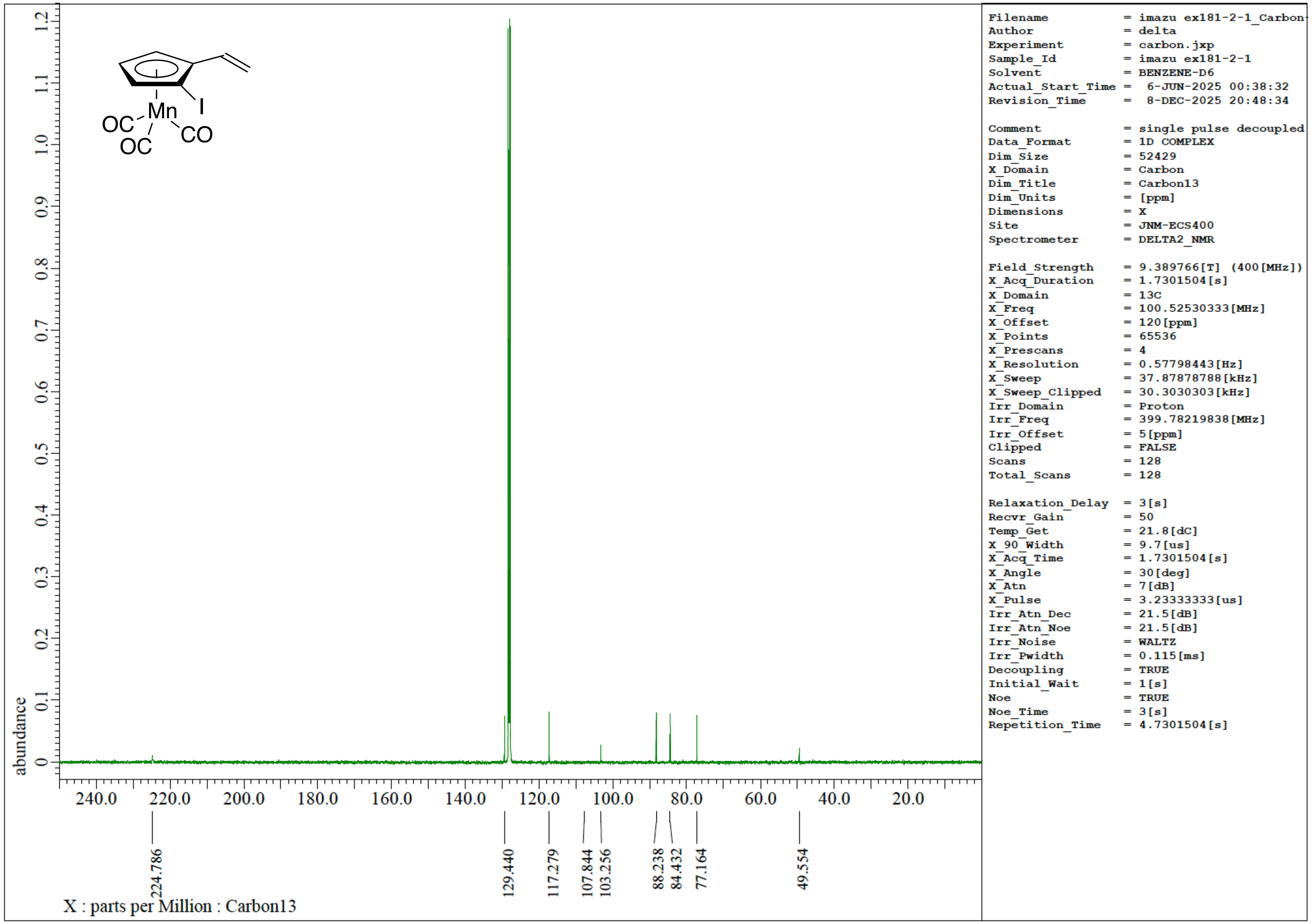

Figure S6. ^{13}C NMR Spectrum of **1c** in C_6D_6 at 101 MHz.

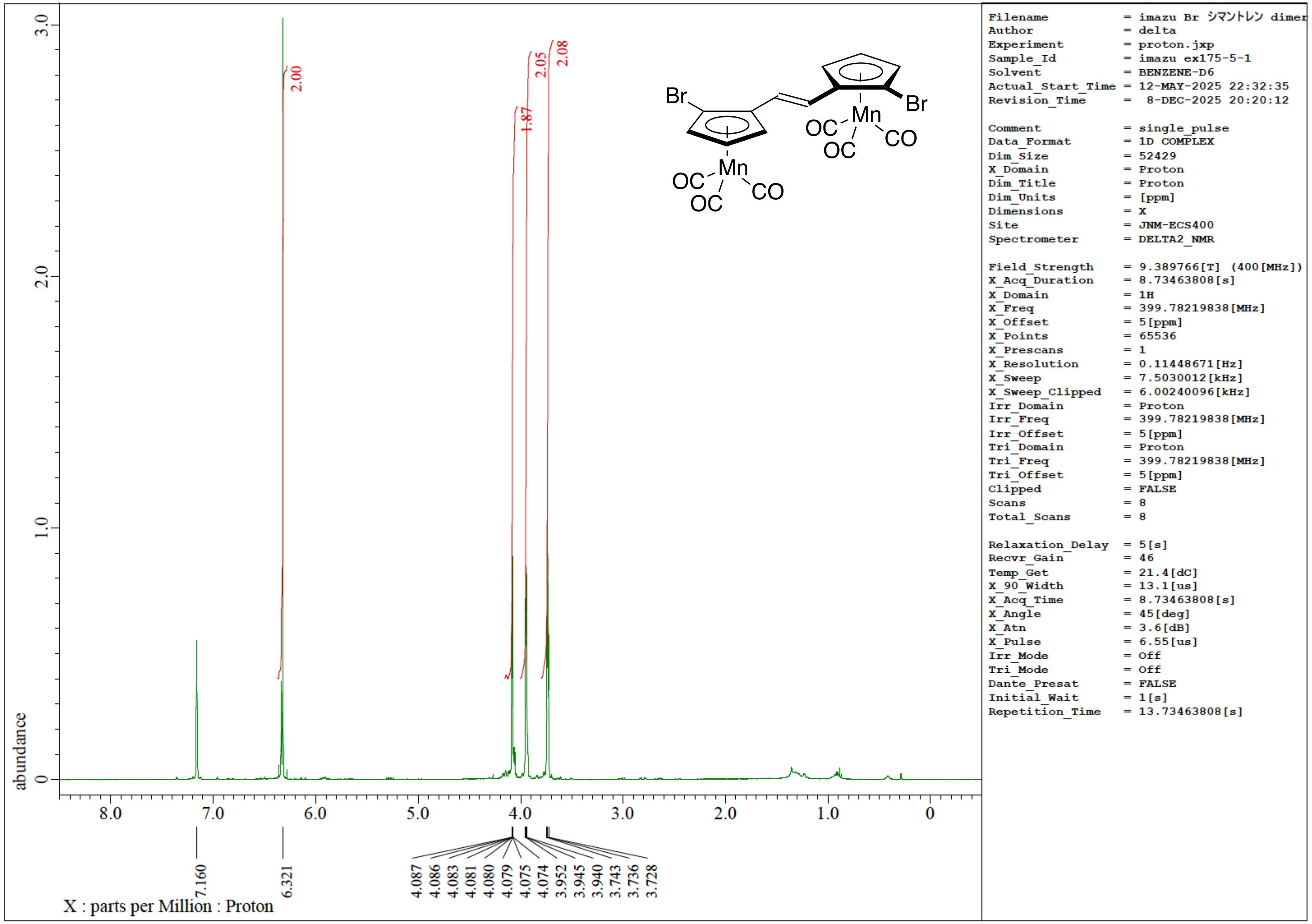

Figure S7. ^{1}H NMR Spectrum of **2a** in C_6D_6 at 400 MHz.

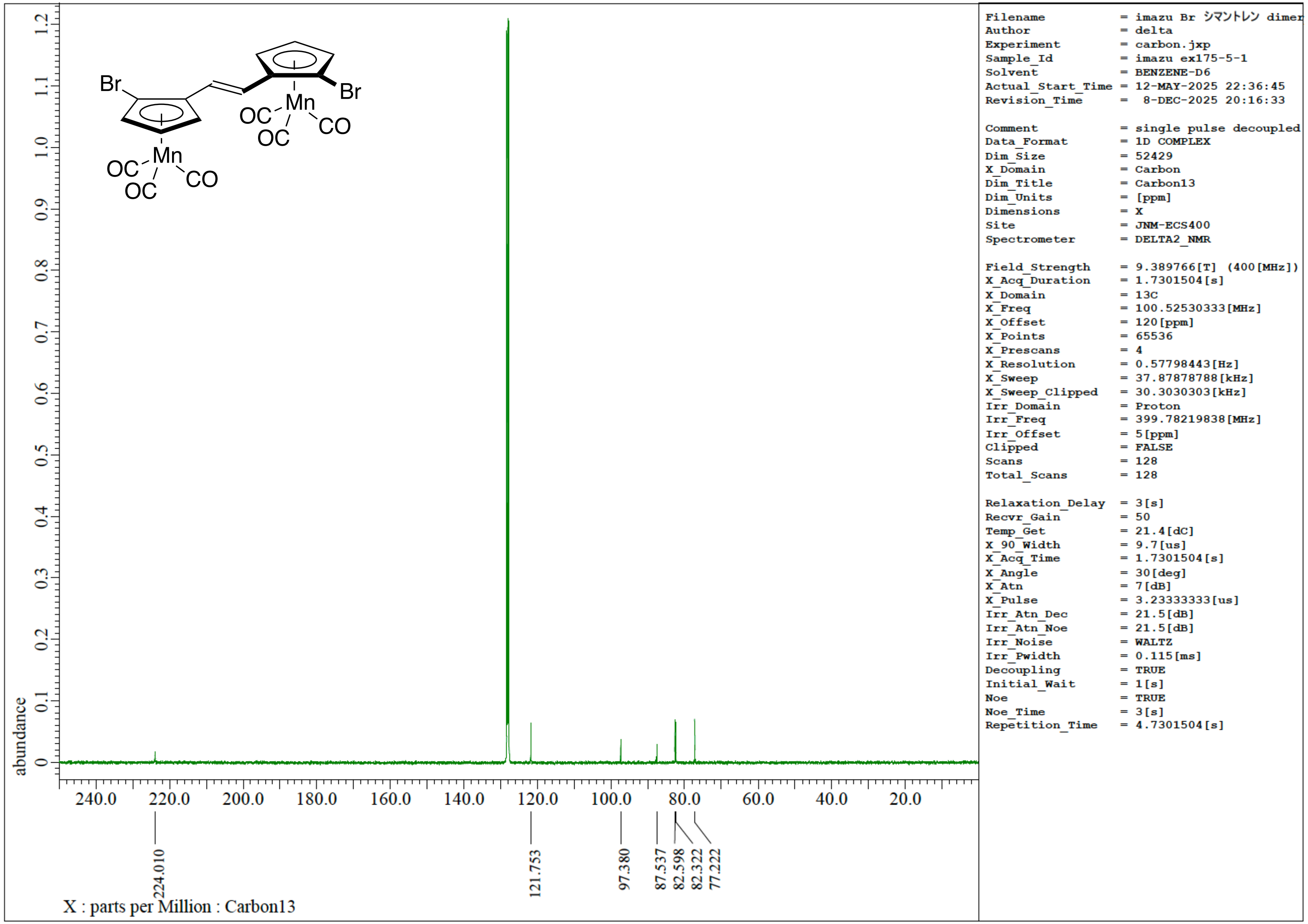

Figure S8. ^{13}C NMR Spectrum of **2a** in C_6D_6 at 101 MHz.

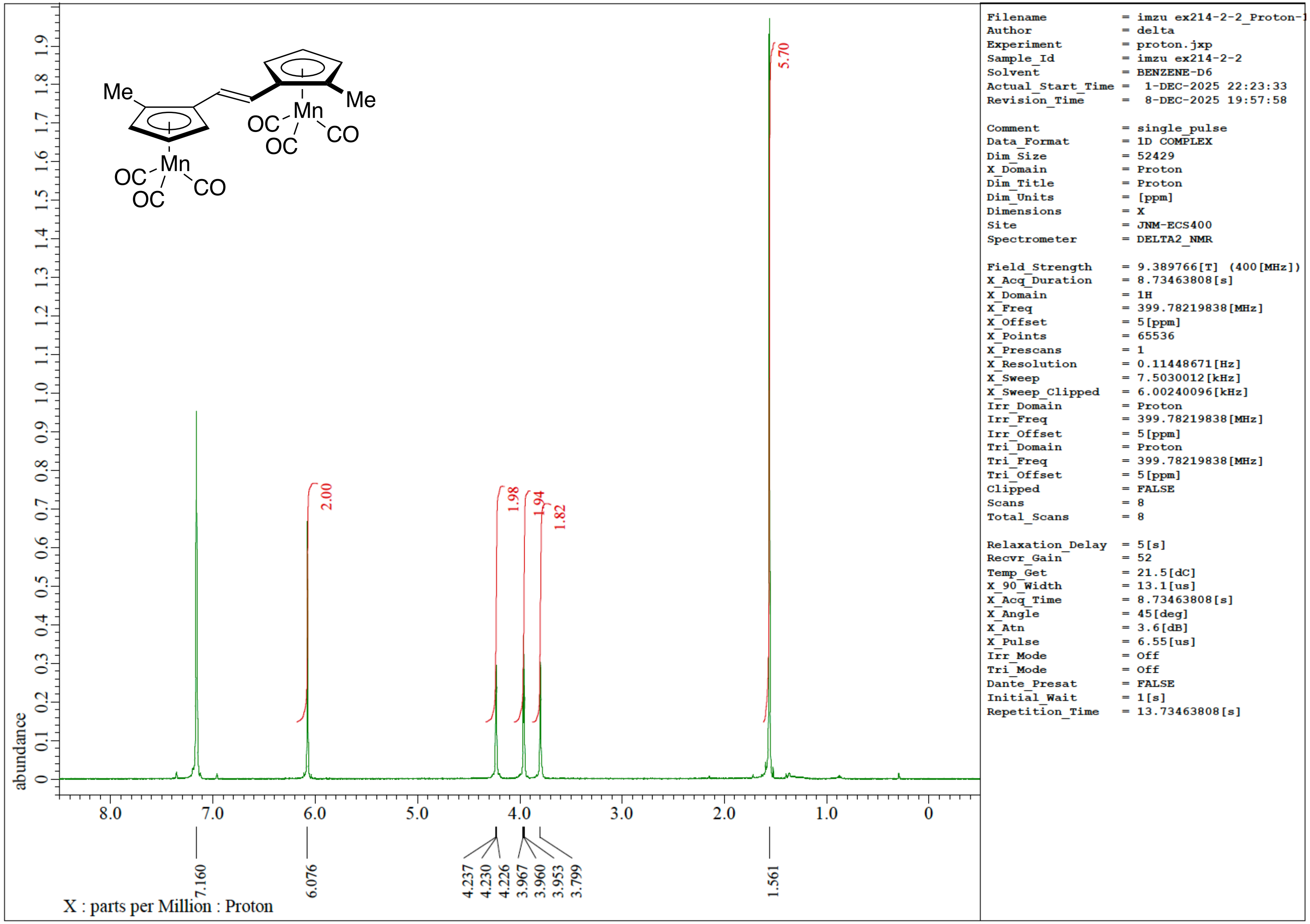

Figure S9. ^{1}H NMR Spectrum of **2b** in C_6D_6 at 400 MHz.

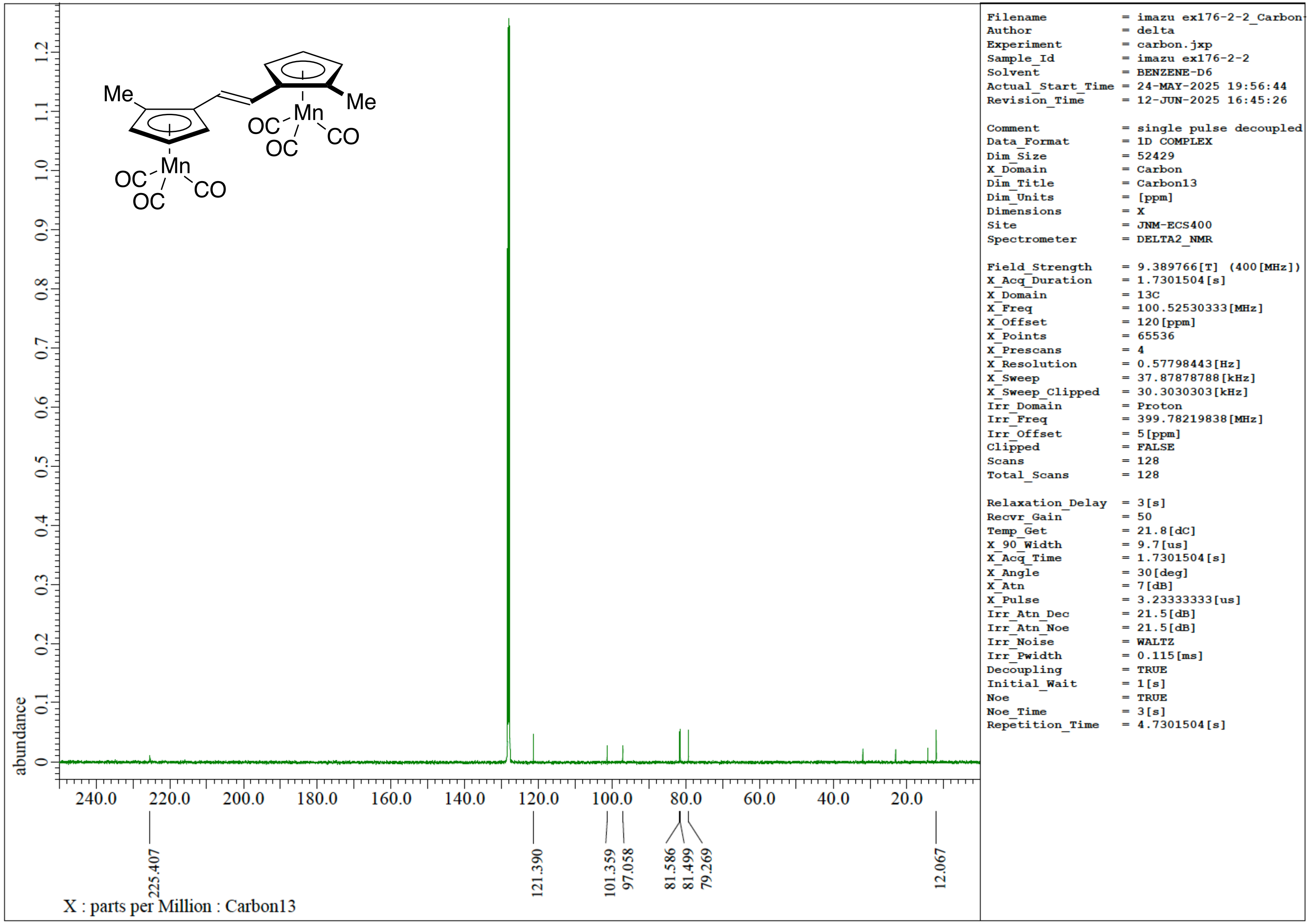

Figure S10. ^{13}C NMR Spectrum of **2b** in C_6D_6 at 101 MHz.

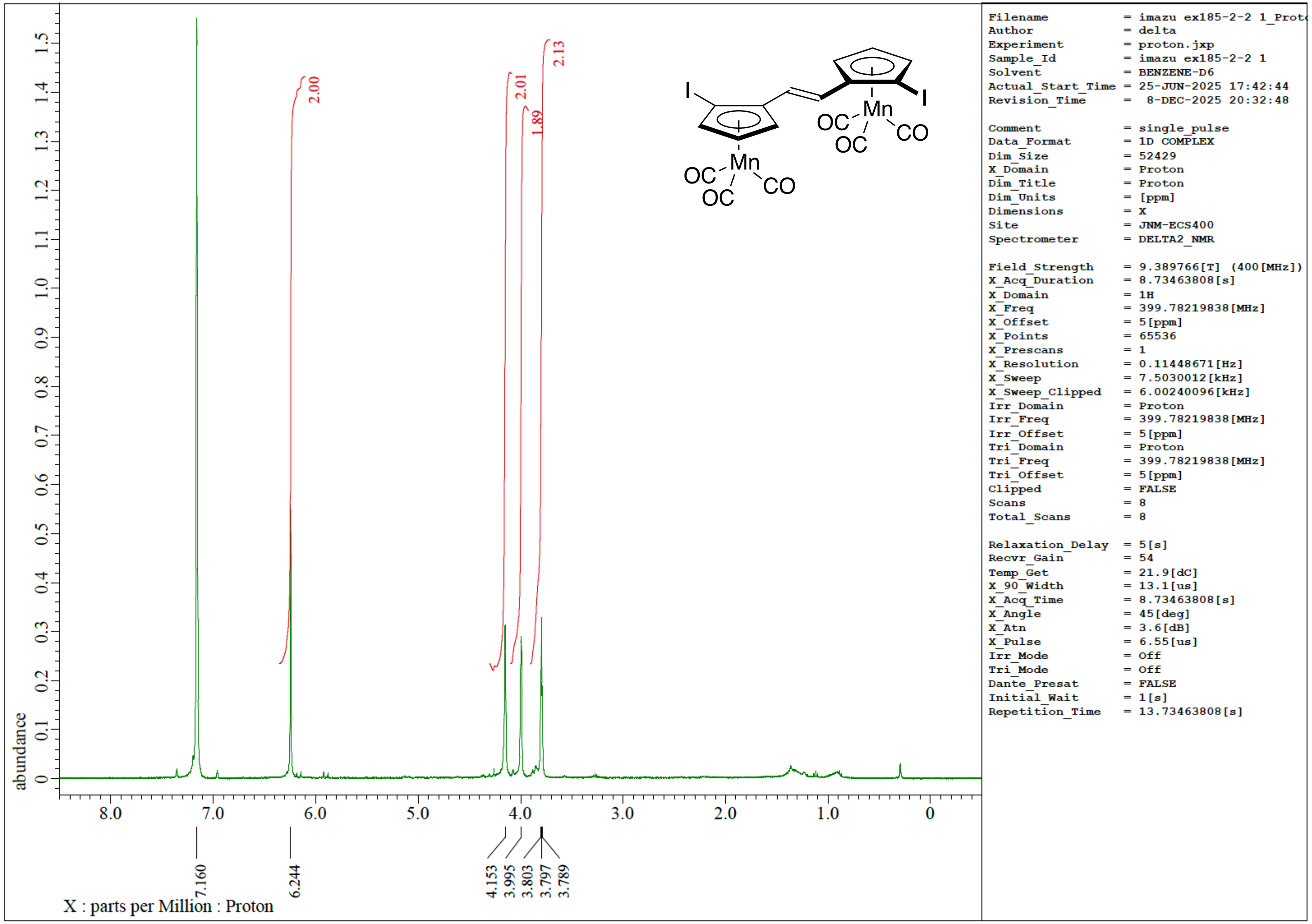

Figure S11. ^{1}H NMR Spectrum of **2c** in C_6D_6 at 400 MHz.

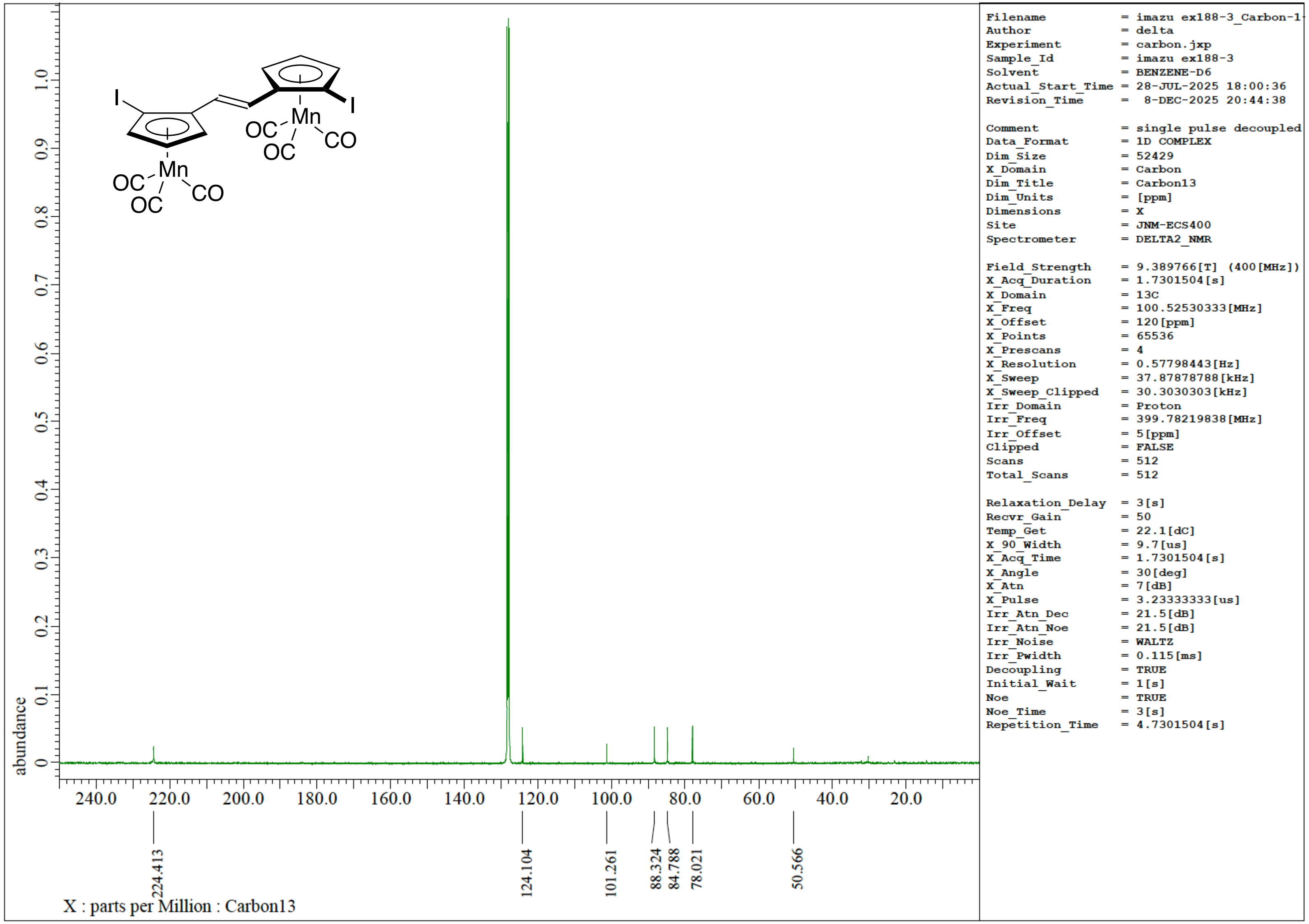

Figure S12. ^{13}C NMR Spectrum of **2c** in C_6D_6 at 101 MHz.

Figure S13. Chiral HPLC Analysis of (*S*)-**1a** (Table 1, entry 4); Chiralcel OD-H; eluent: hexane/^{i}PrOH = 50/1; flow rate: 1.0 mL/min.

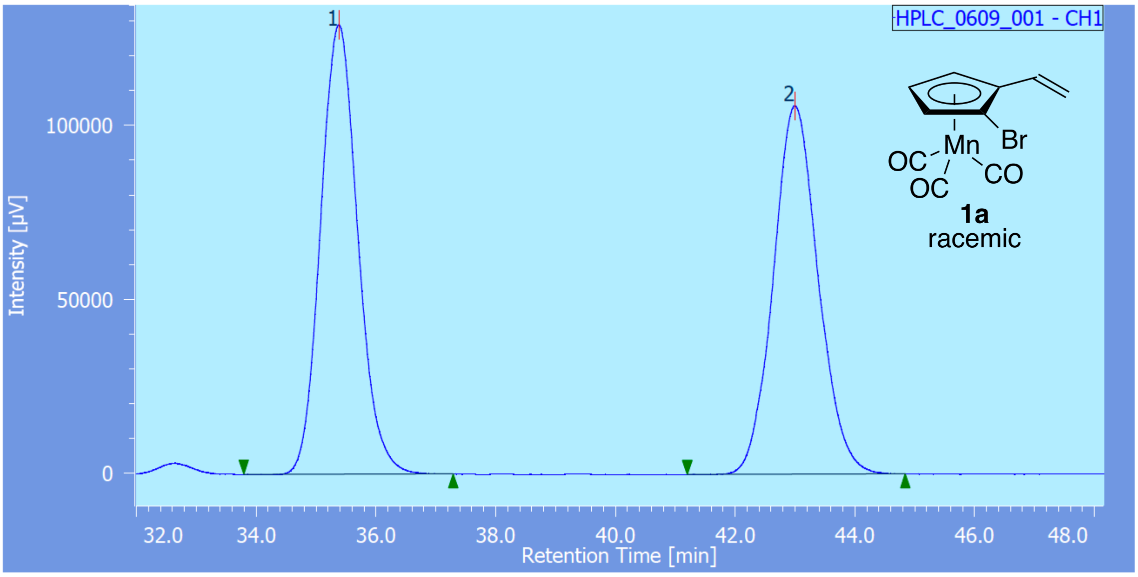

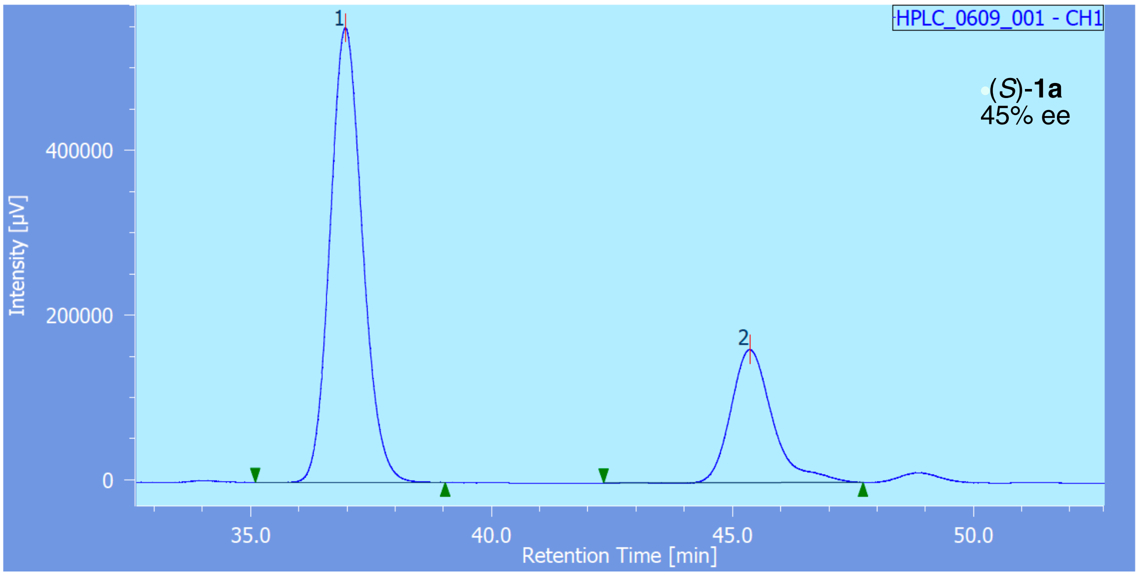

#	peak name	CH	tR [min]	area [µV•sec]	height [µV]	area %	height %	NTP	resolution	symmetry coefficient
1	(*S*)-**isomer**	1	36.967	25817677	551940	72.594	77.353	14618	6.185	1.133
2	(*R*)-**isomer**	1	45.358	9746652	161590	27.406	22.647	14672	N/A	1.426

Figure S14. Chiral HPLC Analysis of (*R*,*R*)-**2a** (Table 1, entry 4); Chiralpak IE; eluent: hexane/ethyl acetate/^{i}PrOH = 90/5/1; flow rate: 0.5 mL/min.

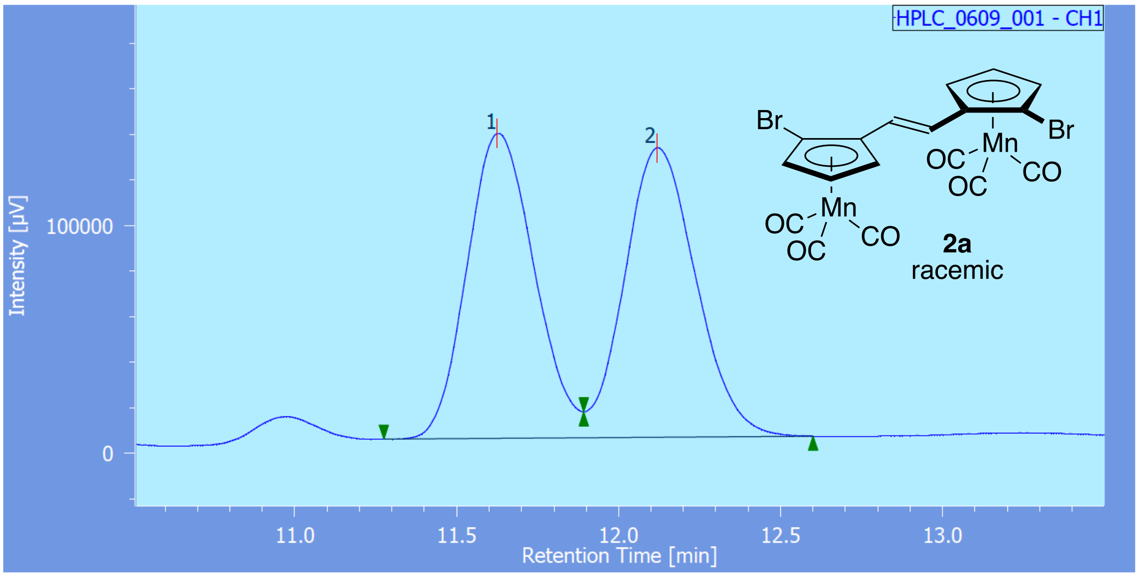

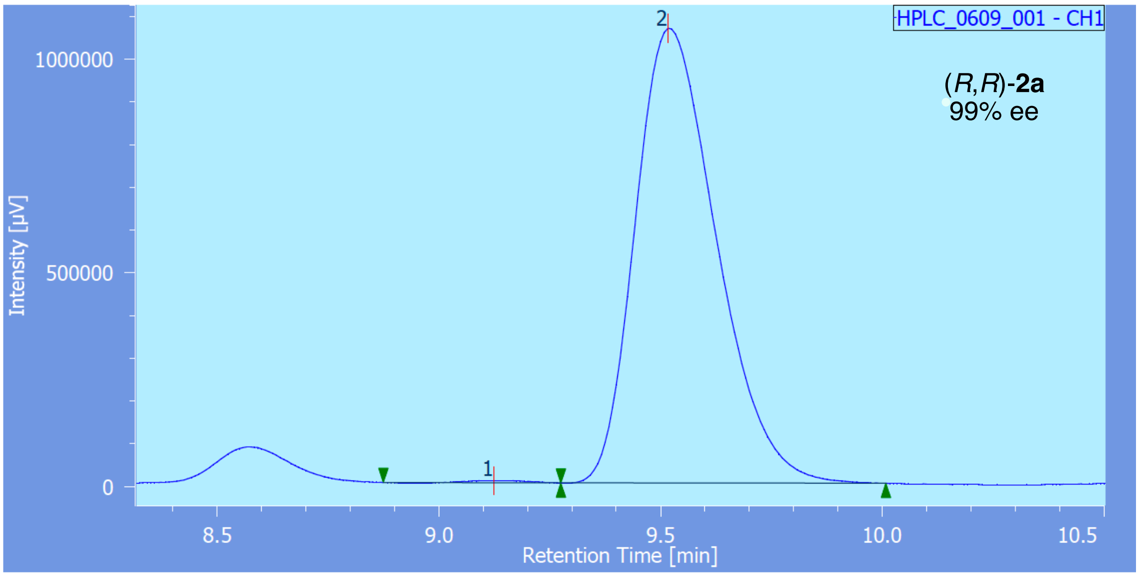

#	peak name	CH	tR [min]	area [µV•sec]	height [µV]	area %	height %	NTP	resolution	symmetry coefficient
1	(*S*,*S*)-**isomer**	1	9.125	45036	4803	0.331	0.450	17571	1.283	1.060
2	(*R*,*R*)-**isomer**	1	9.517	13553796	1063401	99.669	99.550	12787	N/A	1.351

Figure S15. Chiral HPLC Analysis of (*R*)-**1b** (Table 1, entry 8); Chiralcel OD-H; eluent: hexane/^{i}PrOH = 10/1; flow rate: 0.5 mL/min.

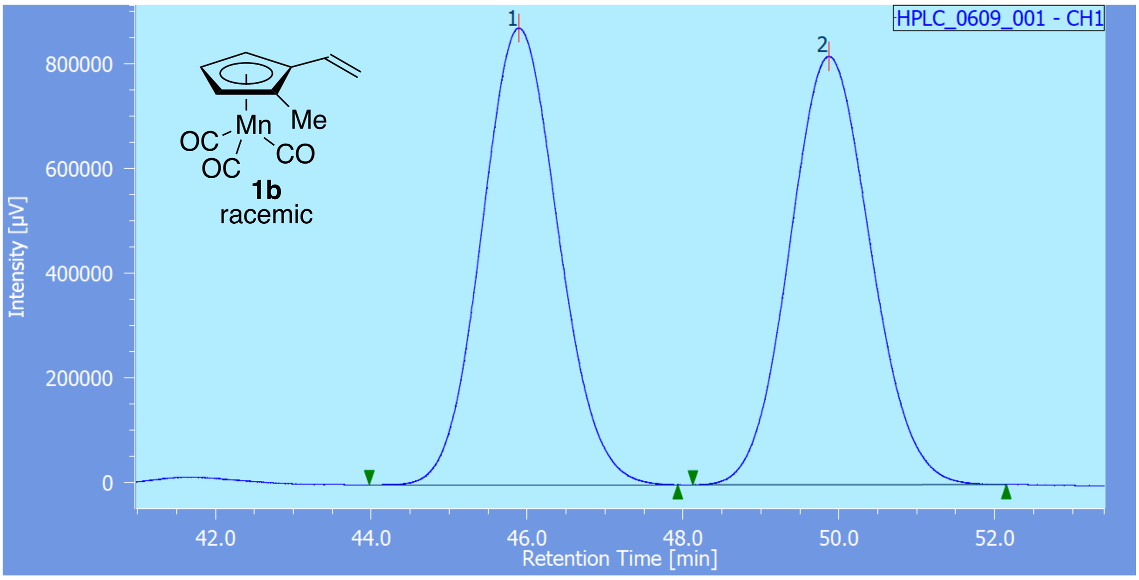

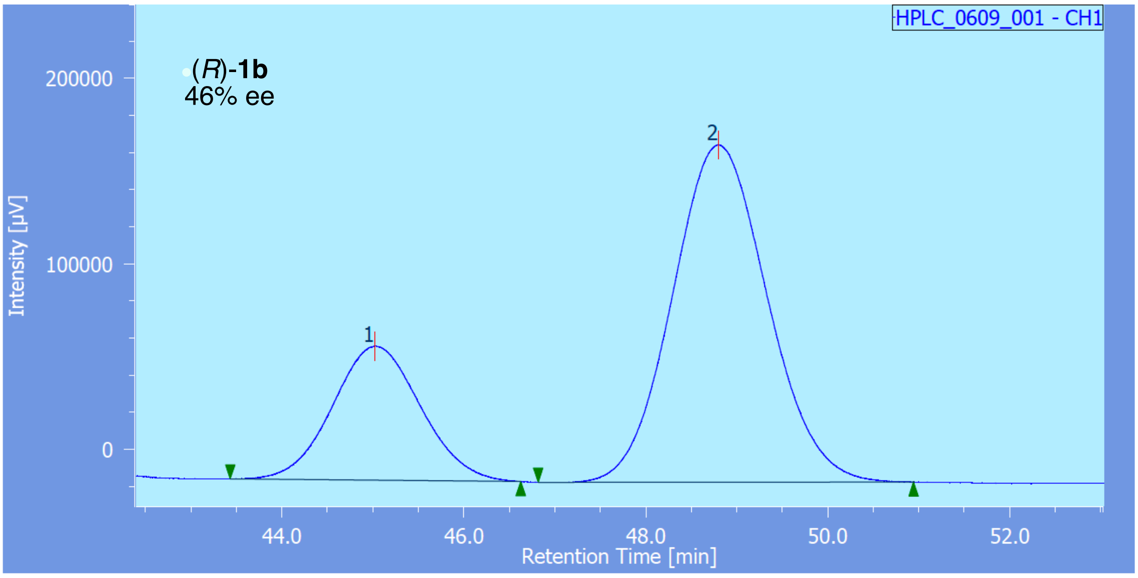

#	peak name	CH	tR [min]	area [µV•sec]	height [µV]	area %	height %	NTP	resolution	symmetry coefficient
1	(*R*)-**isomer**	1	45.025	4867701	72197	26.995	28.428	10211	2.046	1.054
2	(*S*)-**isomer**	1	48.800	13164482	181771	73.005	71.572	10364	N/A	1.073

Figure S16. Chiral HPLC Analysis of (*S*,*S*)-**2b** (Table 1, entry 8); Chiralpak IC; eluent: hexane/^{i}PrOH = 100/1; flow rate: 0.5 mL/min.

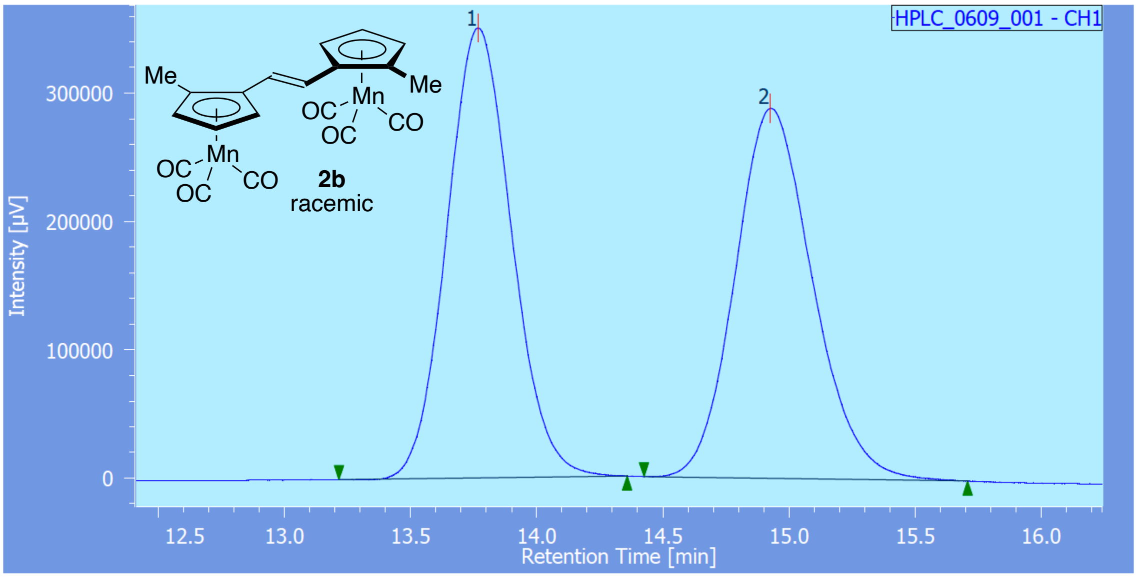

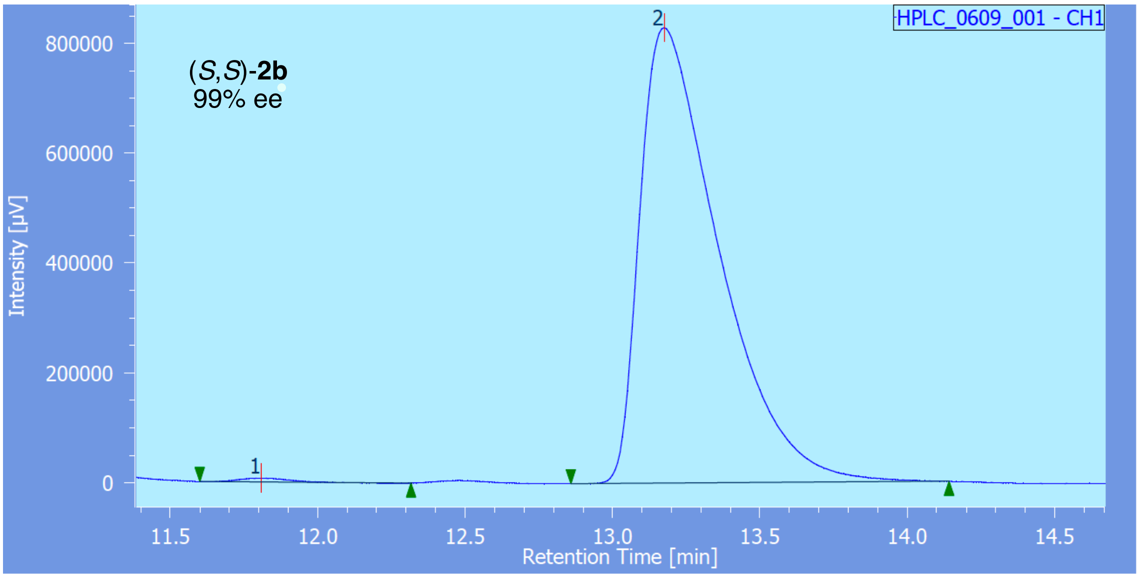

#	peak name	CH	tR [min]	area [µV•sec]	height [µV]	area %	height %	NTP	resolution	symmetry coefficient
1	(*R*,*R*)-**isomer**	1	11.808	84773	7087	0.545	0.846	21349	3.410	1.299
2	(*S*,*S*)-**isomer**	1	13.175	15468192	828833	99.455	99.152	12037	N/A	2.058

Figure S17. Chiral HPLC Analysis of (*S*)-**1c** (Table 1, entry 10); ChiralPAK IB; eluent: hexane/ethyl acetate/^{i}PrOH = 900/60/1; flow rate: 0.5 mL/min.

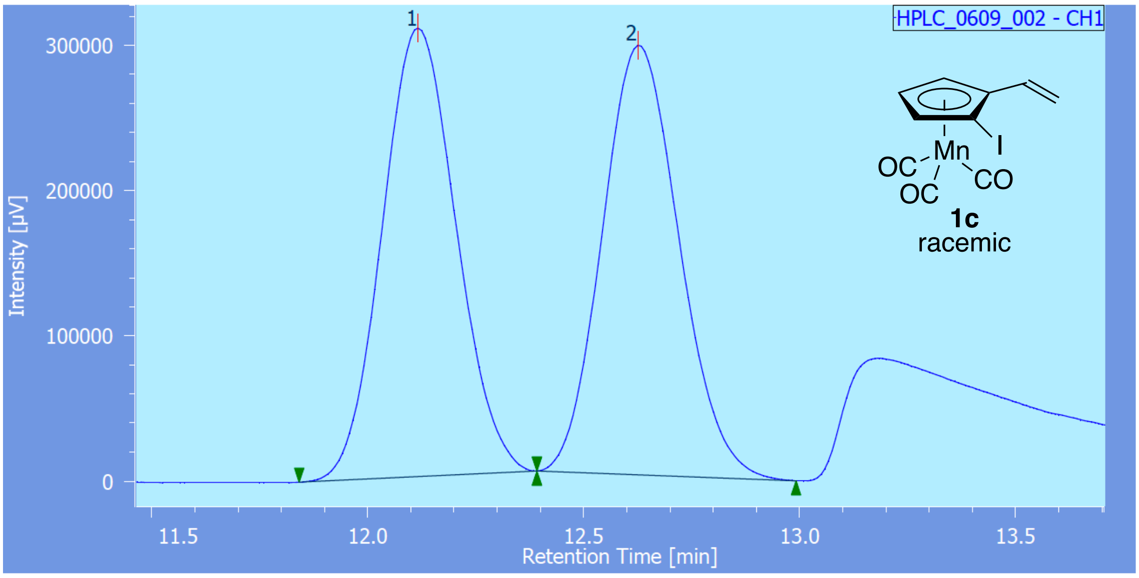

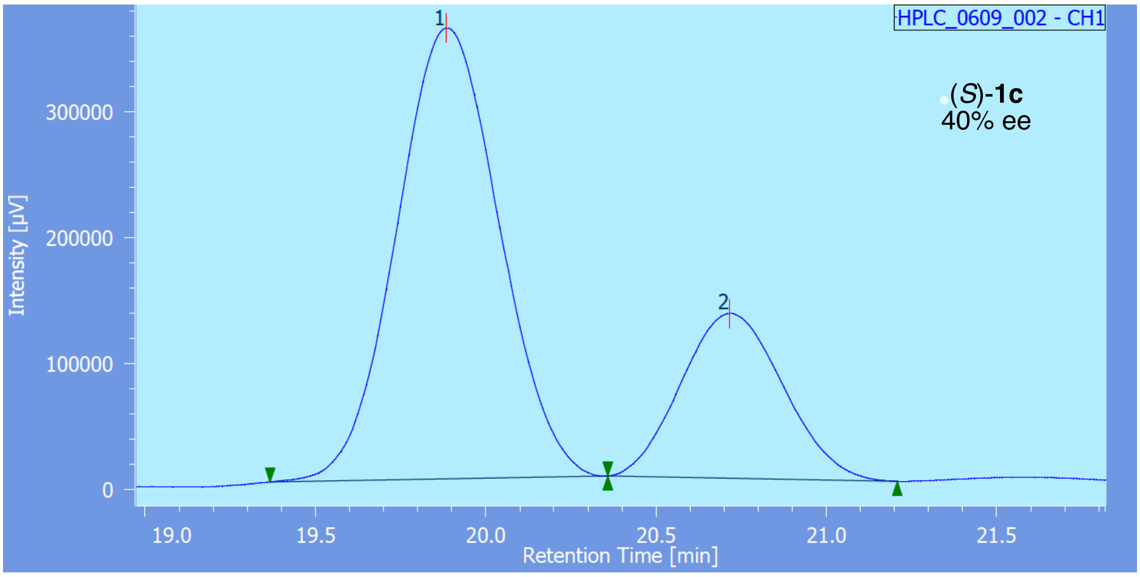

#	peak name	CH	tR [min]	area [µV•sec]	height [µV]	area %	height %	NTP	resolution	symmetry coefficient
1	(*S*)-**isomer**	1	19.883	7496491	358455	73.336	73.250	20179	1.486	1.046
2	(*R*)-**isomer**	1	20.717	2725569	130903	26.664	26.750	21550	N/A	1.089

Figure S18. Chiral HPLC Analysis of (*R*,*R*)-**2c** (Table 1, entry 10); Chiralpak IB; eluent: hexane/ethyl acetata/^{i}PrOH = 90/5/1; flow rate: 0.5 mL/min.

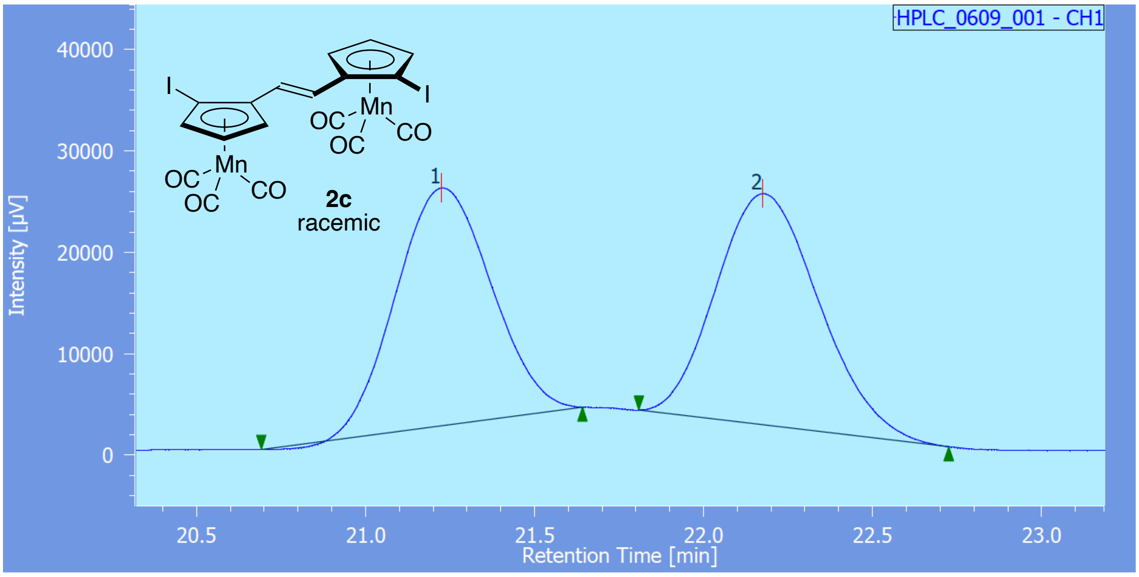

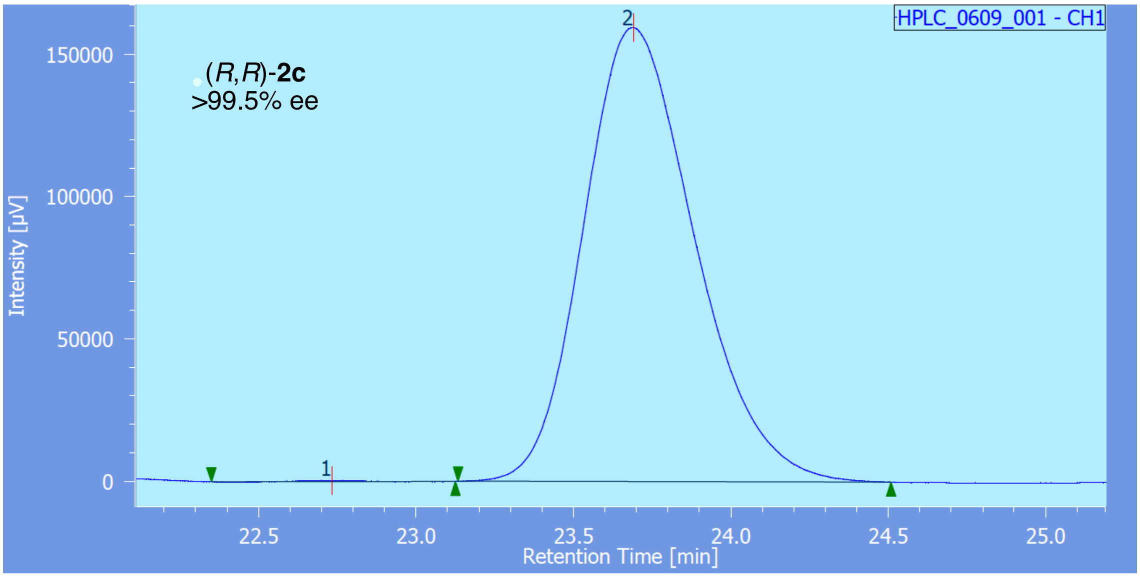

#	peak name	CH	tR [min]	area [µV•sec]	height [µV]	area %	height %	NTP	resolution	symmetry coefficient
1	(*S*,*S*)-**isomer**	1	22.733	5360	400	0.136	0.250	54248	1.854	0.937
2	(*R*,*R*)-**isomer**	1	23.692	3927596	159488	99.864	99.750	21513	N/A	1.209
